# Barriers and facilitators to implementing evidence-based interventions among third sector organisations: a systematic review

**DOI:** 10.1186/s13012-018-0789-7

**Published:** 2018-07-30

**Authors:** Anders Malthe Bach-Mortensen, Brittany C. L. Lange, Paul Montgomery

**Affiliations:** 1 Department of Social Policy and Intervention, University of Oxford, Barnett House, 32 Wellington Square, Oxford, OX1 2ER UK; 2 Department of Social Policy, Sociology and Criminology, University of Birmingham, B15 2TT, Birmingham, UK

**Keywords:** Evidence-based intervention, EBI, evidence-based practice, EBP, third-sector organisation, TSO, Implementation, Barriers, Facilitators, Enablers, Obstructers, Charities, Non-profits, CBO, NGO

## Abstract

**Background:**

The third sector is becoming a growing provider of public, social, and health services. However, there is little evidence on the effectiveness of third sector organisations (TSOs), and their capacity to implement evidence-based interventions (EBIs). Understanding implementation aspects of service delivery remains an important issue in clinical practice, but is poorly understood in the context of TSOs. This is problematic, since implementation issues are known to be critical for effective intervention outcomes.

**Objectives:**

To identify and synthesise existing research on what barriers and facilitators influence the implementation process of TSOs delivering EBIs.

**Methods:**

This review is reported according to PRISMA guidelines and was pre-registered in PROSPERO. Key databases were searched using relevant terms, experts in the field were contacted, and websites were reviewed. All identified studies were double-screened, and data were extracted independently by two authors. Included studies were synthesised using thematic analysis and were quality appraised.

**Results:**

Thirty-one studies were included, most of which were conducted in North America. The thematic synthesis identified *resource limitations*, in particular staff and finance, to be the most reported barrier to TSOs implementing EBIs. *Organisational culture*, including factors such as alignment between the mission of the TSO and EBI, and *support/prioritisation* of the implementation process were the most reported facilitators. These findings generalise across the included studies and are robust to study quality assessment.

**Conclusions:**

While it is often assumed that good outcomes follow when implementing interventions that have been developed and tested according to best practice, little attention has been paid to how EBIs are best transported, contextualised, and implemented by third sector providers. This systematic review found that TSOs faced considerable challenges in implementing EBIs, which were primarily a lack of support and expertise, and unclear/insufficient guidelines on how to adapt EBIs to different populations. To address these challenges, it is important to engage with central stakeholders, such as funders, researchers, policymakers, and practitioners, to discuss how these needs can be met.

**Trial registration:**

PROSPERO: CRD42017073090.

**Electronic supplementary material:**

The online version of this article (10.1186/s13012-018-0789-7) contains supplementary material, which is available to authorized users.

## Background

The third sector is expanding and becoming a growing provider of public, social, and health services in many high-income countries [1–4]. However, little research demonstrates evidence on the *effectiveness* and *impact* of third sector service deliveries [2, 3, 5, 6].

While scholars struggle to agree on a universally applicable definition of the third sector [7, 8], most follow [9], who point to five characterising traits of third sector organisations (TSOs), i.e. that they are (1) formally structured, (2) privately owned and independent from the government, (3) non-profit distributing, (4) self-governing, and (5) benefitting from voluntary activities [5, 10, 11]. However, the use of terminology is variable and terms such as ‘non-profits’, ‘NGOs’, ‘community-based organisations’ (CBOs), ‘charities’, and ‘voluntary organisations’ are often used synonymously. To avoid confusion on the arbitrary distinction between these terms, we will adopt the more commonly applied term ‘third sector organisation’ to denote any such organisation [7, 12].

The third sector is often considered to entail distinct features and characteristics in its service delivery compared to public and private sector bodies [2–5, 13]. For instance, TSOs are thought by some to be better at connecting with hard-to-reach populations, while also being driven by more altruistic values [5, 14, 15]. These perceived traits often appear in the discourse of policymakers, who continue to emphasise the growing importance of the third sector, especially in the context of alleviating social problems [3, 16–19]. Yet, despite the political willingness to increase the role of the third sector in public service provisions, there is little research to demonstrate evidence on the *capacity* and *capability* of TSOs to successfully adhere to evidence-based practice (EBP) and to implement evidence-based interventions (EBIs) [4, 20, 21].

Adherence to EBP implies having established feedback mechanisms between services and outcomes, and the inclusion of stakeholders and the best available evidence in decision-making processes [22–25]. In enabling TSOs to become more evidence-based, there has been a growing emphasis on the development of ‘evidence hubs’ (e.g. What Works Network [26], Project Oracle [27], Blueprints for Healthy Youth Development [28], and Diffusion of Evidence Based Interventions (DEBIs) [29]), which are efforts to develop disseminating databases of interventions and programmes that are ‘proven to work’. A set of proven programmes could enable service providers (both private, public, and third sector) to adopt and implement programmes that are supported by sound scientific evidence with the highest potential for effectiveness. However, while TSOs are known to deliver EBIs in the context of, for example, HIV [30] and addiction [31], there is little systematic research providing a general overview on the types of EBIs implemented by the third sector.

While the principles of EBP are gradually becoming an integrated part of common and accepted practice in social work and clinical settings, practitioners still experience substantial barriers to implementing EBIs [32, 33]. Implementation is often understood in terms of *implementation fidelity*, which can be defined as ‘the degree to which interventions are implemented according to the design.’ [34]. Implementation fidelity is inevitably linked to the concept of *adaptation*, which can be understood as ‘changes made in the original program during implementation (program modification, reinvention)’ [35]. There is an ongoing debate on how practitioners should balance fidelity and adaptation when implementing EBIs, which will be discussed later in this review. Further implementation aspects include adherence to protocol, dose of treatment, participant responsiveness to intervention, and implementation quality [34, 36].

Past research has shown that most intervention studies fail to report adequately on implementation aspects such as fidelity and adaptation, which represent an important blind spot in understanding ‘true’ intervention effects and in conducting meaningful replications [35, 37–39]. The failure to understand aspects related to implementation introduces the risk of overlooking type iii errors (‘implementation failure’) [40, 41], i.e. failure to implement an intervention as intended [42]. Overlooking this issue has great implications for policy and practice, as it may lead to false inferences about the effectiveness of interventions and programmes.

Thus, without understanding the *implementation aspects* of third sector service deliveries, it is difficult to assess their potential to substitute for public sector provision of social and health services. Just as relatively little is known about implementation aspects of EBP in clinical settings [32], there is even more limited research on the capacity of TSOs to implement EBIs. This failure to understand aspects of the implementation ability of TSOs is worrisome, in that such a research gap questions the potential of TSOs to become evidence-based service providers. As the role of the third sector is becoming more salient in the delivery of public and social services, it is critical to ensure that such organisations are willing and able to implement effective and safe services supported by the appropriate evidence-base; especially considering that interventions have the potential to do harm to service-users [43–45]. Further, most TSOs in, for example, the UK work around ‘social services’ and target vulnerable population groups, such as disabled people, children, and the elderly [46], which arguably warrant the use of EBP.

### What has been done?

A range of single studies have investigated the experiences and attitudes of TSOs in implementing EBIs and adhering to EBP [30, 31, 47–49], investigating topics such as barriers and facilitators for TSOs to implementing EBIs [31], perceived needs of TSOs adhering to EBP [50], and the attitudes of third sector practitioners to adopting EBIs [50, 51]. Identified barriers to the implementation of EBIs by TSOs tend to involve factors related to organisational culture, such as staff resistance and organisational setting [31, 52], and factors related to the lack of resources [48]. Facilitators include having established affiliations with research institutions and employing skilled staff [47, 48]. An overarching theme of these studies seems to revolve around the notion that TSOs struggle to implement EBIs and experience serious capacity issues in becoming evidence-based providers.

To date, there has been no systematic attempt to aggregate and analyse existing research on the implementation ability of TSOs delivering EBIs. This constitutes a significant knowledge gap, given that research continues to demonstrate that implementation aspects are critical to the effectiveness of service deliveries [24, 37, 39, 53]. Also, the utility of the increasingly popular ‘evidence hubs’ and ‘blueprints’ relies on the ability of practitioners to *implement* the EBIs according to best practice.

## Objectives

To utilise the full potential of TSOs in the delivery of social and health services, it is crucial to understand what factors influence their implementation process, so that the commissioning and regulation criteria can ensure that delivery is conducted following best practice. The main objective of this review is to aggregate existing research investigating practitioner-identified factors affecting the implementation process of TSOs that deliver EBIs. The focus of the study is captured by the following question:

- What barriers and facilitators influence the implementation process of third sector organisations delivering evidence-based interventions and programmes?

## Methods

To meet these research objectives, a systematic review was conducted following PRISMA guidelines (see Additional file 1 for completed PRISMA checklist) [54]. The protocol was reported according to PRISMA-P guidelines [55] and was pre-registered in PROSPERO (CRD42017073090).

### Search strategy

The search strategy was designed to be exhaustive of the existing literature on studies investigating barriers to, and facilitators of, TSOs delivering EBIs (see Additional file 2 for search terms). A body of research addressing this topic was identified prior to the search through Google Scholar and subsequent reference checking. The bibliography of the pre-identified literature was hand-searched to identify further studies.

The following databases were searched during the systematic review using text words: ABI/INFORM Global, Applied Social Sciences Index & Abstracts (ASSIA), International Bibliography of the Social Sciences (IBSS), MEDLINE®, PAIS Index, Policy File Index, Social Services Abstracts, Worldwide Political Science Abstracts, Social Care Online, SCOPUS, and Open Grey.

The search was revised until it was sensitive enough to capture at least all the pre-identified studies. The final body of included studies was hand-searched for additional references that may not have been captured by the search. Experts in the field were contacted and websites of key organisations reviewed.

### Selection criteria

To be eligible for inclusion, studies had to be primary research or systematic reviews investigating the perspectives and/or experiences of third sector practitioners (e.g. managers, directors or service providers) with regards to the implementation/adoption of EBIs. We considered all studies that investigated the process of delivering an EBI. This process might include, but is not limited to, aspects such as *fidelity* to intervention protocol, and whether *adaptations* were made in the delivery of the intervention. We define EBIs as ‘interventions which have been tested and validated according to the principles of evidence-based practice’. EBIs may be implemented in the context of, for example, social care, healthcare, education, child services, or mental health services, but other contexts of implementation were also considered for inclusion. We define barriers as ‘any factors that obstruct the capacity for third sector organisations to implement evidence-based interventions’. We define facilitators as ‘the factors that enable the implementation of evidence-based interventions’.

All research designs were eligible for inclusion. To be eligible for inclusion, studies had to investigate factors operating as barriers and/or facilitators to the implementation of evidence-based programmes and interventions by TSOs, but this did not need to be the focus of the studies. If it was unclear whether the samples included TSOs, the authors of the studies were contacted for clarification.

Articles were screened at the title and abstract level independently by both ABM and BL using the Rayyan systematic review software [56].

#### Data extraction

All data were independently double-extracted by ABM and BL with the following information being retrieved (see Additional file 3):

- Publication year and author
- Study aim
- Methods (study design, data collection methods, data analysis, and inter-rater reliability)
- Population (type of organisations, area of work, and sample size)
- Types of EBIs implemented
- Results (barriers and facilitators in implementing EBIs)
- Discussion (suggestions for future research and policy)

For qualitative studies, interview and focus-group quotes were double-extracted in separate documents (see Additional file 4: Appendices S1 and S2). These quotes were utilised to extract factors not captured by the individual studies and to evaluate the reliability of the subsequently constructed themes. After finishing the double-extraction, both reviewers (BL and ABM) met in person to review all identified factors, which were discussed until consensus was reached. Upon reaching consensus, the synthesis was initiated.

### Data synthesis

To analyse the included studies, a thematic analysis was conducted, in line with best practice when aggregating data from different types of research [57–60]. Specifically, all identified factors were identified and organised into barriers and facilitators and counted by frequency. The identified factors were then categorised following thematic analysis [60], thus enabling the synthesis to account for the arbitrary difference of factors revolving around the same underlying problem. Identified factors were only counted once per study, except for studies which identified factors specific to different subgroups (e.g. according to different organisational cases [61] or by different types of EBIs [31]). To the best of our knowledge, all included studies investigated unique samples.

The construction of themes was done inductively and entirely according to the factors identified in the data extraction. ABM conducted the full thematic analysis, which was reviewed by BL and PM on an iterative basis. All modifications were made through discussion until consensus was reached.

Additionally, we constructed two tables (Tables 5 and 6) following Rees et al. [62] to provide an overview of how the individual studies contributed to the construction of the identified themes. A study was considered to contribute to a theme if it identified at least one factor part of that theme. This enabled an assessment of whether certain studies were over- or under-represented in the thematic framework [57]. Further, it allowed for assessing the reliability of the identified themes based on an overall judgement of the quality of the studies that contributed to the individual themes. A sensitivity analysis was conducted excluding studies of low quality to test the robustness of findings.

### Quality appraisal

To ensure transparency, all included studies were subject to best practice quality appraisal. For qualitative studies, a modified version of the Joanna Briggs Institute and CASP checklist was applied (Additional file 4: Appendix S3) [63]. For the appraisal of survey studies, a modified version of the AXIS checklist was employed (Additional file 4: Appendix S4). For mixed-methods studies, the appropriate quality appraisal tool was decided according to the type of method employed by the study to identify barriers and facilitators to implementation. These tools allowed for an overall assessment of key biases of the included studies, and the final quality ratings were subsequently utilised to assess the reliability of the identified factors. All appraisals were conducted independently by ABM and BL with any disagreements resolved through discussion until consensus was reached.

## Results

**Fig. 1:**
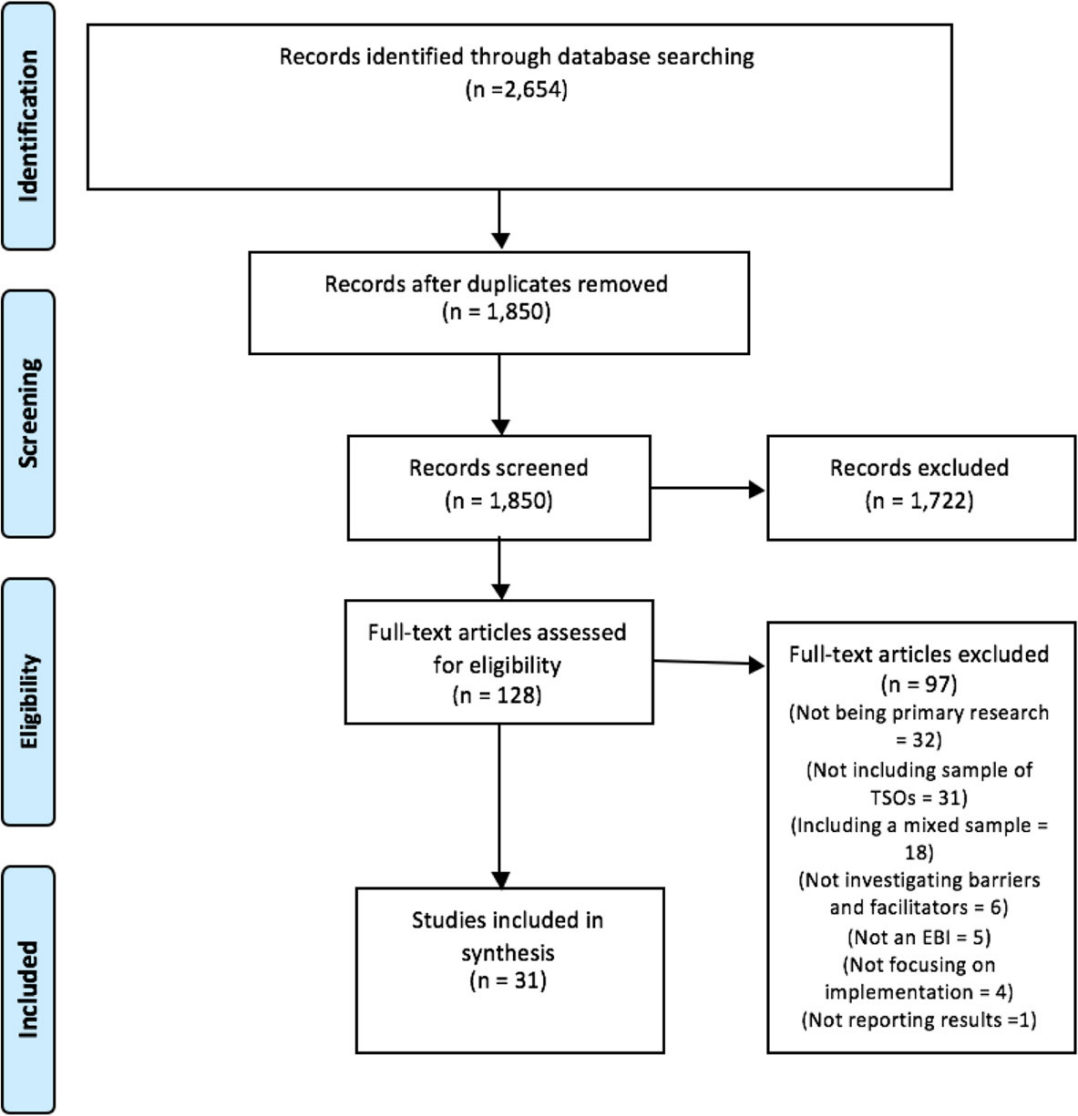
PRISMA diagram

Two thousand six hundred fifty-four articles were identified through the database searches of which 1850 remained after removal of duplicates. One thousand seven hundred twenty-two studies were excluded based on screening titles and abstracts. One hundred twenty-eight studies were reviewed in full text, in which studies were excluded for not being primary research (*n* = 32), not being TSOs (*n* = 31), including a mixed sample of organisations (*n* = 18), not investigating barriers and facilitators (*n* = 6), not focusing on EBIs (*n* = 5), not focusing on the implementation process (*n* = 4), and not reporting sufficiently on the results (*n* = 1). Thirty-one studies were included for the thematic synthesis, which were all identified through the database searches (Fig. 1). No additional studies were retrieved via searches of websites or by reviewing the references lists of the included studies. All studies suggested by the contacted experts or identified via websites that were eligible for inclusion had already been identified by the database searches.

### Characteristics of included studies

All included studies were published after 2009 in peer-reviewed journals, except for two doctoral dissertations [64, 65]. Of the 31 included studies, 26 employed qualitative methods [20, 30, 31, 48, 49, 51, 61, 65–79] and 5 were cross-sectional [50, 80–83].

### Sample size of included studies

**Fig. 2:**
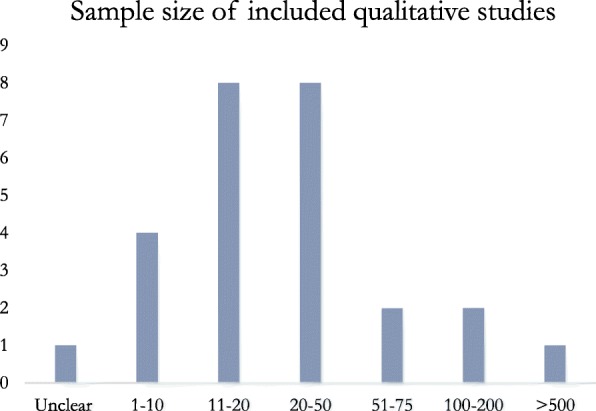
Sample size of included qualitative studies

**Fig. 3:**
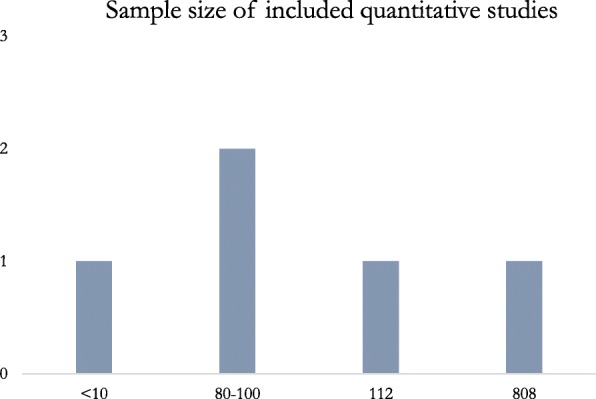
Sample size of included quantitative studies

In the research employing qualitative methods, 4 studies included a sample of 1–10 practitioners [61, 65, 74, 77], 16 studies included a sample of 11–50 practitioners [30, 48, 49, 52, 64, 66, 68, 69, 71–73, 75, 76, 79, 84, 85], 2 studies included a sample of 51–75 practitioners [20, 70], and 3 studies investigated more than 100 participants [31, 51, 67] (Fig. 2). One article did not provide clear information on its sample [78]. For the quantitative articles, 1 study investigated a sample of fewer than 10 TSOs [80], 1 study included 82 TSOs [50], 1 study included a sample of 100 TSOs [50], 1 study included 112 practitioners from 41 NGOs [82], and the last study investigated 510 staff members and 296 directors [81] (Fig. 3).

### Types of organisations

The majority of the included research was conducted in the United States (US) (28/31) [20, 30, 31, 48, 49, 51, 61, 64–67, 69–81, 83–85], with three studies being conducted in Chile [68], Puerto Rico [50], and Australia [82]. Most studies (23/31) referred to the included organisations as ‘community-based organisations’ (CBOs) [20, 30, 31, 48–51, 61, 64, 65, 67, 69–72, 74–76, 78, 80, 81, 83, 85], with five studies using the term ‘non-profits’ [52, 66, 68, 73, 77], two referring to its included sample as ‘churches’ [79, 84] (in the US, churches are considered non-profits), and one study denoting its included organisations as NGOs [82].

### Types of EBIs

All included studies investigated EBIs in the context of health and social outcomes, including addiction [31, 51, 64, 67, 83, 86], HIV [20, 30, 49, 65, 69, 71, 75, 76], exercise [66, 70, 73, 74], social work [68], cancer education [85], parenting [77], nutrition [79, 84], and with a number of studies focusing on mixed types of health EBIs [48, 50, 52, 72, 82, 85] (see Table 1). Nine studies focused on factors influencing the implementation of interventions from the diffusion of evidence-based interventions (DEBI) programme by the Center for Disease Control and Prevention (CDC) [20, 30, 49, 65, 69, 71, 72, 76, 78], and six studies investigated EBIs designed by the Center for Substance Abuse Treatment (SAMHSA/CSAT) [31, 51, 64, 67, 83, 86].

**Table 1:** Types of EBIs investigated in included studies

Types of EBIs	Cited in
Addiction-related EBIs	
– Mixed types of EBIs supported by SAMHSA/CSAT	(Dippolito et al. [67]; Kegeles et al.[86]; Lundgren et al. [47, 51])
– Adolescent Community Reinforcement Approach	(Amodeo et al. [31]; Hunter et al. [83])
– Motivational Interviewing	(Amodeo et al. [31])
– Assertive Community Treatment	(Amodeo et al. [31])
– Cognitive Behavioural Therapy	(Amodeo et al. [31])
– Integrated Dual Disorder Treatment	(Maharaj [64])
HIV-related EBIs	
– Mixed types of DEBIs	(Collins et al. [76]; Gandelman and Dolcini [69]; Dolcini et al. [30]; Owczarzak [71]; Owczarzak and Dickson-Gomez [49]; Veniegas et al. [75])
– The Mpowerment Project	(Kegeles et al. [20])
– ‘The Modelo de Intervención Psicomédica’	(Pemberton [65])
Mixed health-related EBIs	
– EBIs for underserved populations	(Payan et al. [72]; Ramanadhan et al. [48])
– Mixed health EBIs	(Kimber et al. [52])
– EBIs for youth having experienced abuse	(Thomas et al. [82])
– Mixed cancer education EBIs	(Vanderpool et al. [85])
– Unspecified	(Martínez et al. [50])
Exercise EBIs	
– Enhance®Fitness	(Belza et al.et al. [66]; Petrescu-Prahova et al. [73])
– Home-based exercise programme for breast cancer survivors	(Pinto et al. [74])
– Mixed exercise EBIs	(Lattimore et al. [70])
Sexual education	
– Becoming a Responsible Teen (BART)	(Demby et al. [61])
– Teen pregnancy-prevention EBIs	(House et al. [80])
– ‘Cuicidate’	(Feutz and Andresen3 [78])
Nutrition focused EBIs	
– ‘Body and Soul’	(Allicock et al. [84])
– Mixed EBIs on increasing vegetable and fruit consumption	(Honeycutt et al. [79])
Social work	
– Unspecified	(Flores et al.[68])
Parenting EBIs	
– ‘Together Facing the Challenge’	(Murray et al. [77])

### Quality of studies

Overall, 29 of the included studies were rated to be of either high or medium quality, with only three studies being rated as low quality. Of the 26 included qualitative studies (see Additional file 5), nine were rated to be of high quality, 15 of medium quality, and two of low quality. Of the five included survey studies (see Additional file 6), four were rated to be of medium quality, and one study to be of low quality.

### Barriers and facilitators

The synthesis identified 80 unique factors operating as barriers across 31 studies and 57 factors operating as facilitators over 24 studies. Table 2 illustrates the five most reported factors operating as barriers and facilitators to implementation of EBIs. The most reported barriers were related to recruitment and retention of service-users (14/31), problems in adapting EBIs (13/31), lack of financial resources (13/31), lack of staff resources (11/31), and implementation difficulty (9/31).

**Table 2:** Top five most reported factors operating as barriers and facilitators

Top 5 cited barriers and facilitators	
Barriers	Facilitators
Recruitment/retention issues 14/31	EBI matches well with mission of TSO 9/24
Problems in adapting the EBI 13/31	Flexibility regarding the implementation of interventions 9/24
Lack of financial resources 13/31	Perceived effectiveness of EBI 8/24
Lack of staff resources/high staff turnover 11/31	Organisational support/prioritisation of EBI6/24
Implementation difficulty/fidelity issues 9/32	Supportive leadership 5/24

The most reported facilitating factors were related to whether the EBI matched with the mission of the TSO (9/24), flexibility for TSOs to implement the EBI (9/24), perceived effectiveness of the EBI (8/24), organisational support and prioritisation of the EBI, and supportive leadership (5/24).

### Thematic analysis

The main results of the synthesis are illustrated in Tables 3 and 4, which display the prevalence of identified factors organised according to their underlying theme. All identified factors were given a reliability rating to reflect how consistently each factor was reported on and the quality of studies identifying it (see ‘Key for reliability ratings’ row).

**Table 3:** Themes of factors operating as barriers

Themes	Times cited	Reliability of findings
Resources	51	
Lack of financial resources	13	High reliability
Lack of staff resources/high staff turnover	11	High reliability
Lack of time	8	High reliability
Insufficient space for all services	5	High reliability
Resources (unspecified)	5	High reliability
Lack of resources for training	3	High reliability
Lack of scientific resources	3	Medium reliability
Lack of technical resources	3	Medium reliability
Client and community factors	37	
Recruitment/retention issues	14	High reliability
Client resistance and non-participation	5	High reliability
Difficult population	5	High reliability
Lack of community support/community resistance	4	High reliability
Client receptiveness and commitment	3	High reliability
Client (individuals and family) attendance is poor	2	High reliability
Community resources (including substance abuse treatment) are lacking	2	High reliability
Stigma	1	Low reliability
Anonymity issues	1	Low reliability
Delivery capability	33	
Implementation difficulty/fidelity issues.	9	High reliability
Lack of expertise/experience	7	High reliability
Lack of administrative infrastructure	3	High reliability
Cultural/language barriers	3	High reliability
Competing responsibilities	2	Medium reliability
Supervision issues	2	Medium reliability
Programme needs	2	Medium reliability
Delivering the EBI in a rural area	1	Medium reliability
Scheduling challenges	2	Low reliability
Strategic planning	1	Low reliability
Balancing needs between of the CBO and research	1	Low reliability
Organisational culture	32	
Conflict with EBI and organisational identify/mission/culture	6	High reliability
Staff resistance	5	High reliability
Lack of prioritisation of the EBI/org support	3	High reliability
Lack of preparation of staff to become evidence based providers	3	High reliability
Lack of leadership	3	Medium reliability
Incomplete buy-in from organisation	2	Medium reliability
Resistance to change (keeping status quo)	2	Medium reliability
Change in staff and leadership	2	Medium reliability
Lack of EBI champions	1	Medium reliability
Lack of belief in the efficacy of the intervention	1	Medium reliability
Unclear mission of the organisation	1	Medium reliability
Higher level of stress	1	Medium reliability
Low cohesion in the organisation	1	Medium reliability
EBP devaluing existing practices by the TSO	1	Low reliability
Adaptation issues	22	
Problems in adapting the EBI	13	High reliability
Lack of fit between EBI and target population of TSO	7	High reliability
Conflict between the EBI and expert knowledge of the provider	1	Low reliability
Not knowing how to communicate adaptation issues	1	Low reliability
Training capacity issues	19	
Staff not trained well enough/ Training provided for the EBI not adequate	8	High reliability
Lack of EBI training	4	High reliability
Variance in staff training and perspectives	2	Medium reliability
Lack of training available	1	Low reliability
Training new staff	1	Low reliability
Programmes were not packaged for training	1	Low reliability
Conducting community needs assessments	1	Low reliability
Proposal development	1	Low reliability
Monitoring issues	10	
Not having monitoring practices in place to evidence effectiveness.	4	High reliability
Data management issues	2	Medium reliability
Interoperability challenges between systems	1	Medium reliability
Issues around evaluation and monitoring of implementation	3	Low reliability
Intervention-specific barriers	10	
Transportation	3	High reliability
State education law in conflict with EBI	1	Low reliability
State law prohibits collection of certain data	1	Low reliability
State law requires approval by school board and parental review committee	1	Low reliability
Assessing light versus moderate activity	1	Low reliability
Incorporating physical activity into the participants’ lifestyle	1	Low reliability
Geographically dispersed offices made consistent implementation difficult	1	Low reliability
Logistical issues	1	Low reliability
Commissioning requirements	8	
Certification process was burdensome/time consuming	3	High reliability
Lack of support by funder	2	Medium reliability
Prohibition on adaptation by funders.	1	Medium reliability
Funder demand for major modifications to EBI	1	Low reliability
Funder rejection of modifications to EBI	1	Low reliability
Collaboration issues	6	
Hard to establish collaboration agreements	2	Medium reliability
Lack of strategies for community mobilisation	1	Low reliability
Lack of integration of services	1	Low reliability
Hard to find potential academic partners	1	Low reliability
Assistance in strategies for the development of EBIs	1	Low reliability
Others	3	
Competition with other afterschool activities	1	Low reliability
Development and analysis of public policies	1	Low reliability
Inability to fund programme partners because of local agency policies	1	Low reliability
Key for reliability ratings^*^		
High reliability	The identified factor is consistently supported by several studies of medium quality and from one high-quality study, or the study is supported by at least two high-quality studies.	
Medium reliability	The identified factor is supported from several medium-quality studies, or the factor is identified from at least one high-quality study.	
Low reliability	The identified factor is supported by several studies of low quality and/or single studies of medium quality.	

^*^It should be noted that confidence in findings do not relate to the *generalisability* of the findings

**Table 4:** Themes of factors operating as facilitators

Themes	Times cited	Reliability of findings
Organisational culture	40	
EBI matches well with mission of TSO	9	High reliability
Organisational support/prioritisation for EBI	6	High reliability
Champions	4	High reliability
Supportive leadership	5	Medium reliability
Inclusive organisational team/culture	4	Medium reliability
Staff motivation to deliver EBI	3	Medium reliability
Organisational capacity	3	Medium reliability
Strategic planning	2	Medium reliability
Organisational stability	1	Medium reliability
Internal flexibility of resources	1	Low reliability
Involving staff and volunteers	1	Low reliability
Strength of planning committee	1	Low reliability
Delivery capability	18	
Staff expertise	4	High reliability
Receiving intervention training	4	High reliability
Staff experience with delivering EBI	4	Medium reliability
Administrative infrastructure and capacity	4	Medium reliability
Continuous training	1	Low reliability
Clear staff roles in implementing the EBI	1	Low reliability
Accountability	15	
Perceived effectiveness of EBI	8	High reliability
Continuous evaluation of EBI	3	High reliability
Novelty of EF	1	Medium reliability
Relative advantage of EBI	1	Low reliability
Cost effective	1	Low reliability
Using EBI will improve professional practice	1	Low reliability
Adaptation	13	
Flexibility regarding the implementation of interventions	9	High reliability
Match with the target population	4	High reliability
Collaboration/external factors	10	
Invitation to partner with another organisation to offer EF	1	Medium reliability
Working with partner with experience delivering the EBI	2	Low reliability
Working with experienced partners	2	Low reliability
Working with partners with consistent access to target population	1	Low reliability
Partnerships (unspecified)	1	Low reliability
Political support	1	Low reliability
Coordination and communication with other agencies	1	Low reliability
External support	1	Low reliability
Clients/community factors	9	
Use of incentives	2	Medium reliability
Access to target population through existing programmes	2	Low reliability
Experience working with population	2	Low reliability
Ability to recruit and retain IDU clients	2	Low reliability
Client participation in goal setting and feedback.	1	Low reliability
Funders	7	
Access and availability of training	2	Medium reliability
Continuous support in delivering the EBI.	2	Medium reliability
Using EBI that is recognised by funder	1	Medium reliability
Funding stability	1	Low reliability
Flexibility of prioritisation of resources	1	Low reliability
Other	7	
Logistics	1	Low reliability
Established national organisation	1	Low reliability
Communication	1	Low reliability
HIV testing success	1	Low reliability
No. of clinicians certified/employed at grant end	1	Low reliability
No. of supervisors certified/employed at grant end	1	Low reliability
No. of youth served during grant period	1	Low reliability
Resources	4	
Availability of technical assistance	2	Medium reliability
Financial support	1	Medium reliability
Supplying needed resources	1	Low reliability
Intervention specific factors	3	
Easy to use	1	Low reliability
Availability of manuals	1	Low reliability
Implementation is rewarding	1	Low reliability
Key for reliability ratings^*^		
High reliability	The identified factor is consistently supported by several studies of medium quality and from one high-quality study, or the study is supported by at least two high-quality studies.	
Medium reliability	The identified factor is supported from several medium-quality studies, or the factor is identified from at least one high-quality study.	
Low reliability	The identified factor is supported by several studies of low quality and/or single studies of medium quality.	

^*^It should be noted that confidence in findings do not relate to the *generalisability* of the findings

The most prevalent themes impeding implementation revolved around resources (e.g. lack of time, finances, and staff), followed by client and community factors (e.g. recruitment/retention issues), delivery capability (e.g. lack of expertise), and organisational culture (e.g. conflict with EBI and TSO mission). Other significant themes included challenges around adapting the EBI (e.g. not knowing how to adapt the intervention to the target population) and lack of training. Less significant themes involved monitoring issues, intervention-specific problems (e.g. transportation and legal barriers), commissioning requirements (e.g. paperwork), and collaboration issues.

The thematic synthesis of factors operating as facilitators identified the main category to be organisational culture, which involved factors such as organisational support and alignment between the organisational mission and the EBI. Other significant themes included delivery capability (e.g. staff expertise), accountability (e.g. perceived effectiveness of the EBI), and adaptation (e.g. flexibility and match with target population). Less reported factors involved collaboration (e.g. with academic and experienced partners), client/community factors (e.g. community support), funders (e.g. continuous support), resources, and intervention-specific factors (e.g. availability of manuals).

### Robustness of findings

To assess the consistency of the identified factors and the quality of the studies reporting them, all factors were given a reliability rating (see ‘Key for reliability rating’). Further, to ensure that the findings were not driven by the quality of the included research, we re-conducted the thematic analysis excluding the studies of low quality [77, 78, 80], which did not result in any important changes to the thematic categories or the ranking of top cited barriers and facilitators.

Tables 5 and 6 demonstrate how each study contributed to the identified themes. For both barriers and facilitators, the most reported themes were also the ones most representative of the included research. However, there were several inconsistencies between the representativeness of the themes and how often certain categories were reported on. For example, the second most reported category ‘factors related to client and community issues’ was identified in fewer studies than the third and fourth most reported categories. Further, the third most reported category ‘factor related to delivery capability’ was more representative of the included research than the second most reported category. Similarly, for facilitators the second, third, and fourth most reported categories represented roughly the same amount of studies.

**Table 5:**
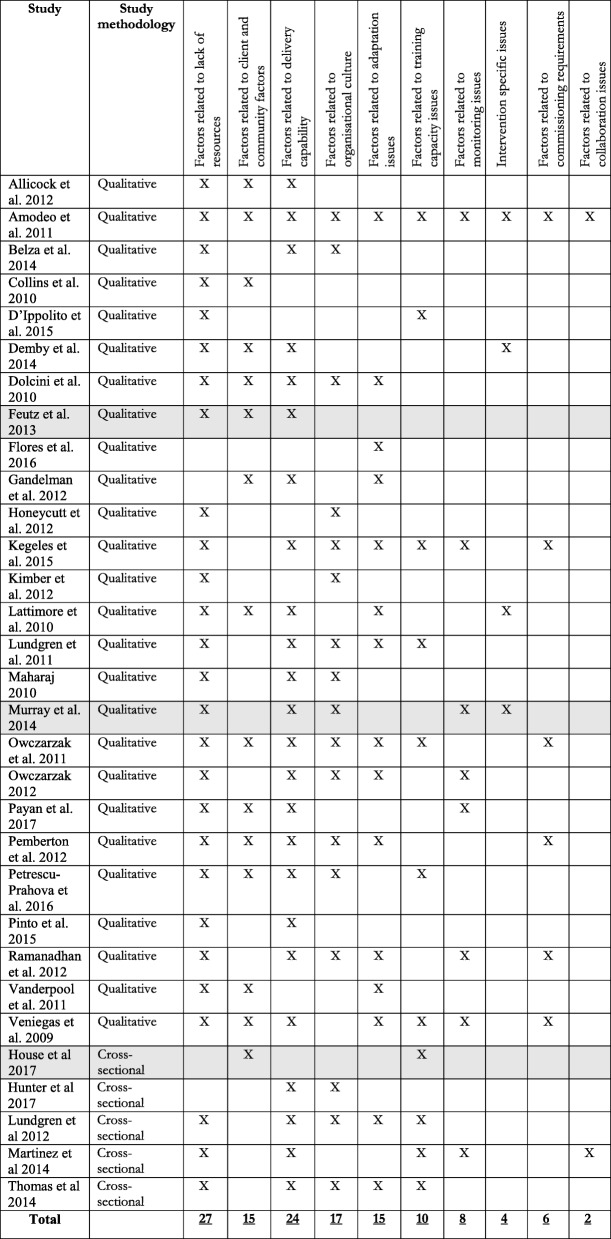
Overview of the contribution of individual studies on the identified themes of barriers

Please note that each study can contribute with multiple factors to the same theme. Shading indicates studies that were rated to be of low quality.

**Table 6:**
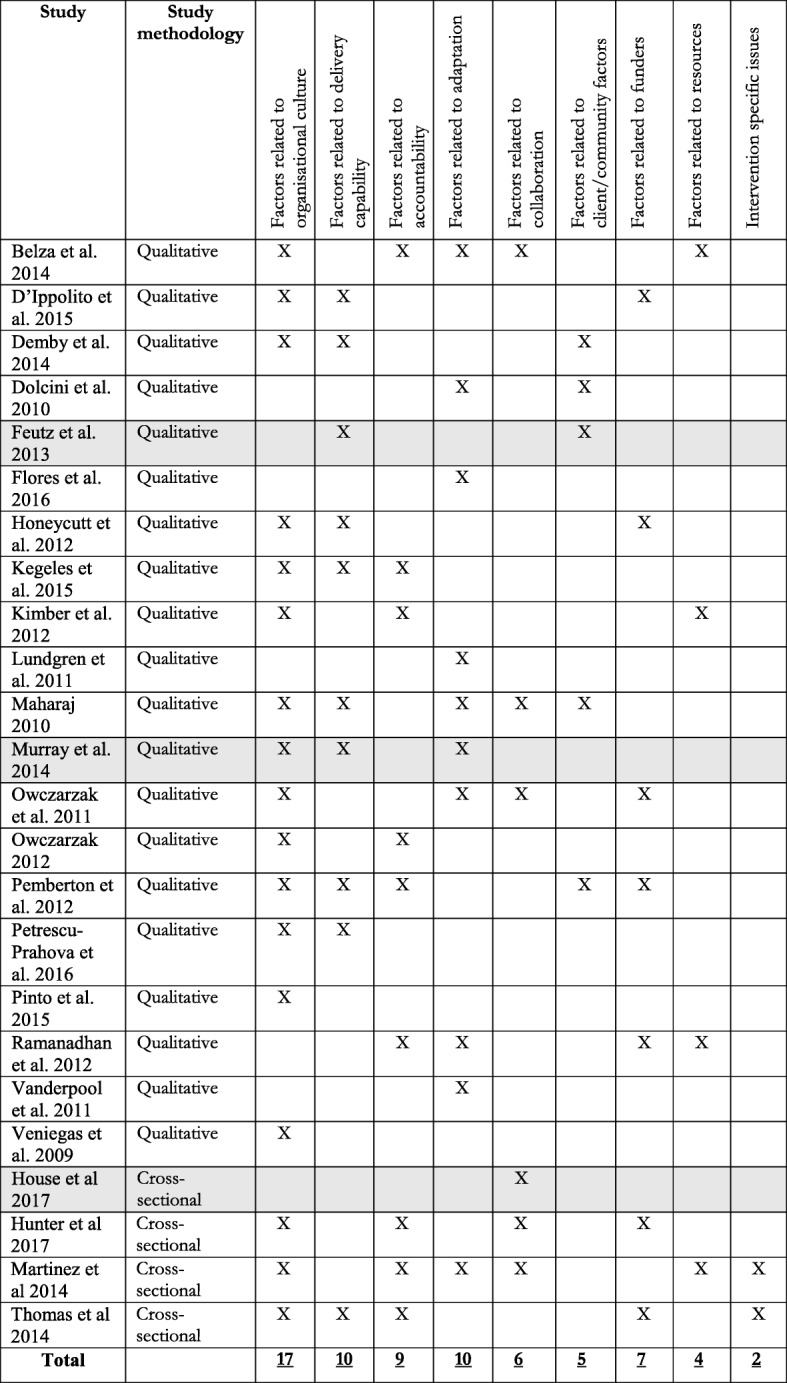
Overview of the contribution of individual studies on the identified themes of facilitators

Please note that each study can contribute with multiple factors to the same theme. Shading indicates studies that were rated to be of low quality.

There was no clear pattern to suggest that study quality or methodology affected what thematic categories to which the included studies contributed.

### Recommendations for policy and practice

Additional to the analysis of factors operating as barriers and facilitators, we also aggregated the recommendations posed by the included research. The main identified themes were split between recommendations targeted at: (1) funders and collaborators, (2) research and practice, and (3) TSO practitioners. The full results can be found in Table 7, and less salient recommendation factors can be found in Additional file 4: Appendix S5.

**Table 7:** Main recommendation categories of the included research

Main themes	Elaboration
Recommendations for funders and collaborators	
Invest in training and capability building	Recommendation cited in 2 studies of high quality, 4 of medium quality, and 2 of low quality.
Assess organisational infrastructure before funding	Recommendation cited in 2 studies of high quality and 2 of medium quality.
Assess fit with organisational mission	Recommendation cited in 2 studies of high quality and 1 of medium quality.
Planning and communication of costs before implementation	Recommendation cited in 2 studies of medium and high quality.
Ensure partnership with community of implementation	Recommendation cited in 2 studies of medium and high quality.
Assess organisational needs of TSOs	Recommendation cited in 2 studies of medium and high quality.
Provide continuous evaluation and support	Recommendation cited in 2 studies of medium and high quality.
Recommendations for research and practice	
Clearer guidelines to how an intervention can be changed to respond to agency and client needs.	Recommendation cited in 2 studies of high quality and 3 of medium quality.
Better understand how TSOs can appropriately modify/adapt interventions	Recommendation cited in 2 studies of high quality and 3 of medium quality.
Request guidance on adaptation versus fidelity by developers.	Recommendation cited in 2 studies of high quality and 1 of medium quality.
To enable and support CBOs in being able to adapt and tailor interventions.	Recommendation cited in 2 studies of medium and high quality.
Recommendations for practitioners	
Ensure necessary capability to implement and adapt	Recommendation cited in 3 studies of high quality and 3 of medium quality.
Invest in organisational infrastructure	Recommendation cited in 3 studies of medium quality.
Preparation to implement	Recommendation cited in 2 studies of medium and high quality.
Maintain and build expertise	Recommendation cited in 2 studies of medium and high quality.

The main recommendations for *funders* are to invest in technical assistance and capability training for the TSOs they fund and to assess organisational infrastructure and ability of TSOs to implement EBIs before offering funding. The main recommendations for *research and practice* are to have clearer guidelines on how to adapt and modify EBIs to different populations and to conduct more research on how EBIs can be modified without compromising effectiveness. The main recommendations for *practitioners* are to ensure capability to implement and adapt EBIs, and to invest in organisational infrastructure. These results will be addressed further in the discussion.

## Discussion

### Summary of findings

This systematic review identified, quality appraised, and synthesised 31 studies, most of which were conducted in the US. The thematic synthesis identified the most reported barrier for TSOs to implement EBIs to revolve around *resources*, in particular with respect to lack of staff and finance. The most reported category of facilitators related to *organisational culture* and included factors such as alignment between the mission of the TSO and the EBI, and organisational support for implementing the EBI. The findings were largely representative of the included studies and robust to study quality.

### Implications for policy and practice

In interpreting the thematic categories, one may distinguish between *capacity* and *capability.* Capacity can be thought of as ‘how much’ an organisation can do and include aspects related to resources and infrastructure, whereas capability can be understood as ‘how well’ an organisation can do and thus relates to issues such as expertise and experience [87, 88]. Importantly, the review demonstrated that TSO practitioners experience central issues related to both capacity and capability. Specifically, the most salient barriers were related to a lack of resources, issues with clients and community (particularly in terms of recruitment and retention issues), and a lack of delivery expertise and experience.

These barriers were also reflected in the recommendations, which highlighted a need for funders to better assess TSOs before providing funding and, when having made the decision to fund an organisation, to provide continuous training and support for that organisation. For practitioners, the main recommendation was to invest in the necessary organisational infrastructure and to ensure that organisations have the technical expertise to implement an EBI. This suggests that funders often do not provide the necessary support for TSOs to implement EBIs, but also that more emphasis should be put on selecting the TSOs that are most capable and motivated to becoming evidence-based providers.

A reoccurring theme for barriers, facilitators and recommendations was that of *adaptation*, in which support for adaptation of EBIs was mentioned as an important facilitator and the lack of adaptation guidelines as a central barrier. Many EBIs, such as DEBIs, come in packages with clear guidelines on how they should be implemented, but do not always address the issue of adaptation and modification [89–91]. Many studies reported that the *one-size-fits-all* form of EBIs often constituted a challenge, particularly regarding being unable to appropriately adapt an intervention to the target population of the TSO. This challenge is, for example, mirrored by the most reported impeding factor being ‘recruitment/retention issues’. Further, several studies reported that TSOs did not receive sufficient training and support in learning how to appropriately adapt the interventions. Some of the challenges around adaptation are reflected in the following quote:

"The question of fidelity is something that they talked about a lot… the boxed [DEBI] interventions are great, but what people really need is more technical assistance about how to effectively adapt these interventions while retaining the theoretical core. They [the agency] needs to build their capacity to understand the internal logic of the M-group piece of the intervention so that they can say ‘here is the logic of this activity, and the behavior it is seeking to address…here is our target population for this intervention…how do we change M-groups for this target population while retaining fidelity to the original design?" [20]

Past research has already emphasised the importance of theorising and outlining the ‘core components’ of interventions as part of implementation guidelines [92, 93]. However, even in clinical practice, appropriate and sufficient reporting on intervention components remains an issue [38, 94, 95]. While efforts are arguably being made to improve the reporting of interventions (e.g. through the TIDieR checklist [96]), this is a fairly recent development, and many EBIs, such as DEBIs, do not always include sufficient descriptions of intervention components [90]. Also, simply reporting on intervention components is not enough; efforts must also be made to consider whether, and if so, how, different components can be effectively adapted to different contexts and populations [97].

The challenge of EBIs sometimes being too inflexible in their form was also mirrored in the recommendations for research and practice, which proposed to better understand how one can effectively modify interventions and to provide clearer guidelines on how to adapt EBIs to different populations. Importantly, this does not mean to suggest that one should strive for *standardisation* of intervention components (e.g. activities), but rather that more efforts are needed to conceptualise how intervention functions or mechanisms can be tailored to different implementation settings [98].

### Ways forward

This systematic review identified the categories reported as barriers and facilitators to be largely distinct, rather than reciprocal which is often the case in this type of review [59, 60, 99]. For example, the most reported barrier, resources, was one of the least reported factors operating as a facilitator. This finding may appear curious, considering that the included studies seem to put heavy emphasis on the lack of resources as a barrier, yet ‘more resources’ was not often identified as a facilitating factor. Similarly, by far the most reported facilitating category, organisational culture, was only the fourth most reported theme of categories operating as barriers.

One may think about this non-reciprocal relationship between barriers and facilitators through the scope of sufficient and necessary factors. That is, to improve practice, it may be necessary to provide more resources for implementation, but it may not be a sufficient step in isolation. As demonstrated by the analysis of facilitators, demonstrating internal motivation to prioritise the EBI was perceived central for successful implementation. However, regardless of the degree of organisational motivation and support, an organisation must have sufficient resources in place to become an evidence-based provider.

The idea that one must consider the implementation process in its entirety was also reflected in the list of recommendations, which entailed different suggestions for different stakeholders. Past research has consistently reported that TSOs are dependent on the requirements posed by funders and commissioners [100–102], and that their ability to adhere to best practice is largely a function of those criteria. Thus, to improve current practice it is central to ensure that commissioning criteria are relevant and facilitating of the best possible practice.

While an increasing body of academic and policy literature contends that the third sector plays an important role in the future delivery of care and health, there seems to be limited attention to how the role of TSOs can be best facilitated. For example, little is known about whether TSOs require specific support in the delivery of EBIs compared to other types of providers. However, TSOs often report that the commissioning requirements imposed on them favour a New Public Management line of thinking, thus focussing on financial reporting and management [103, 104], which, in isolation, may overlook central aspects related to implementation and effectiveness.

To respond to the challenges identified in this review, it is important to consider perspectives from the full system of stakeholders, including practitioners, commissioners, service-users, and policymakers to discuss how TSOs can be best supported in delivering social and health services. To do so, one may draw on stakeholder inclusive methods such as consensus meetings and Delphi panels [105, 106] to determine what types of support TSOs require to appropriately deliver EBIs. Importantly, this would also allow for identifying the organisational *needs* and *constraints* of TSOs and to engage in dialogue about how the commissioning process may better assess the potential of organisations to deliver EBIs and what types of support will best enable successful implementation.

### Limitations

Most of the included studies were conducted in North America, and the findings of this review should be interpreted in light of that context. Similarly, the review aggregated factors from a mixed sample of organisations within health and social care and thus mainly applies to organisations of those types. Further, the review suffers from the fundamental limitation that different factors may affect the implementation of different organisations or EBIs differently. However, the studies which did investigate barriers and facilitators across different EBIs tended to identify factors that revolved around similar themes [31, 61, 76].

The synthesis was done by aggregating identified factors and thus followed a sort of ‘vote-counting’. However, by analysing and categorising the identified factors thematically, the findings should be more robust to factors revolving around the same underlying concept [107]. Also, by mapping the contribution to the categories of the included studies, the review demonstrated how representative the constructed themes were of the included research. Further, all identified studies were quality appraised, and the reliability of the identified factors were assessed according to both the consistency with which they were reported and the quality of studies from which the factors originated.

### Further research

This review focused on the perspectives of practitioners following the assumption that their experiences were closest to the implementation process, but future research may consider a larger group of stakeholders, such as commissioners and policymakers. The review demonstrated that many implementation issues were grounded in the lack of adaptation expertise, driven in turn by the lack of support and expertise, and unclear/insufficient guidelines on how to adapt EBIs to different populations. To address this challenge, future research should investigate how to provide clearer guidelines for TSOs to adapt EBIs and what types of support the adaptation process requires. However, such investigations mandate careful thought on and clear outlining of what types of modifications can be made without compromising core intervention components.

This review focused on factors influencing implementation and did not discuss how these factors might correspond to other aspects, such as *evaluation* [108]*.* Becoming an evidence-based provider does not just entail implementing services developed using the best available evidence and stakeholder preferences [109], but also mandate *continuous* monitoring and evaluation [110, 111]. However, if an intervention is developed and tested according to best practice, is it then necessary to allocate further resources to evaluation? One might argue that requiring continuous evaluation on interventions which have already proved efficacious takes away valuable resources from *service delivery*. To approach this discussion, one might consider the example of mass deworming programmes, which involve medicating children against certain soil-transmitted infections in endemic areas [112]. These programmes have been—and still are—heavily implemented following an assumption of effectiveness based on single studies demonstrating promising results [113]. Yet, the utility of these interventions were recently questioned by two high-quality systematic reviews which both demonstrated null effects of the deworming intervention [114, 115]. Such findings emphasise the importance of continuous evaluation, especially for interventions that have only proved efficacious in single studies. However, to develop recommendations and guidelines on how and when implementation might influence effectiveness, more research is warranted to better theorise how implementation may moderate intervention effects and how commissioning criteria may consider such moderations.

## Conclusion

While it is often assumed that good outcomes follow when implementing interventions that have been developed and tested according to the principles of EBP, little attention has been paid to how EBIs are best transported to different service settings [116]. Generally, past research has arguably taken the aspect of implementation for granted, even in clinical settings [37, 117–119]. However, particularly little is known about the implementation of third sector service deliveries, which is a central blind spot considering the increasing role of TSOs in the delivery of social and health services.

This review constitutes the first systematic attempt to aggregate and analyse the factors that influence the implementation process of TSOs. In so doing, this review addresses the often-implicit assumption that interventions and programmes supported by rigorous evidence can be effectively implemented without considering the implementation ability of service providers. The findings illustrate that TSOs face many capacity and capability issues, which are primarily driven by a lack of support and expertise. Going forward, it is central to involve and engage with stakeholders to discuss how the commissioning process may better identify capable TSOs and offer the necessary implementation support.

## Additional files

Additional file 1: Contains a completed PRISMA checklist. (DOC 62 kb)

Additional file 2 Contains the search strategy employed by the study. (PDF 51 kb)

Additional file 3: Contains the extracted data. (XLS 5724 kb)

Additional file 4: Contains the appendices referred to throughout the study. (DOCX 124 kb)

Additional file 5: Quality of qualitative studies. (DOCX 95 kb)

Additional file 6: Quality of survey studies. (DOCX 91 kb)

## Abbreviations

CBO: Community-based organisations
EBI: Evidence-based intervention
EBP: Evidence-based practice
TSO: Third sector organisation
US: United States

## References

[1] Despard MR (2016). Challenges in implementing evidence-based practices and programs in fit human service organizations. J Evidence-Informed Soc Work.

[2] Dickinson H, Allen K, Alcock P, Macmillan R, Glasby J. The role of the third sector in delivering social care. NIHR School for Social Care Research. 2012; doi:978–0–85328-443-7

[3] Macmillan R (2010). The third sector delivering public services: an evidence review. TSRC Working Paper 20.

[4] Wilson M, Lavis J, Guta A (2012). Community-based organizations in the health sector: a scoping review. Heal Res Policy Syst.

[5] Hardwick R, Anderson R, Cooper C (2015). How do third sector organisations use research and other knowledge? A systematic scoping review. Implement Sci.

[6] Chapman T, Brown J, Crow R (2008). Entering a brave new world? An assessment of third sector readiness to tender for the delivery of public services in the United Kingdom. Policy Stud.

[7] Alcock P (2010). A strategic unity: defining the third sector in the UK. Volunt Sect Rev.

[8] Defourny J, Kirsten Grønbjerg B, Lucas Meijs B, Marthe Nyssens B, Naoto Yamauchi B, Grønbjerg K (2016). Voluntas symposium: comments on Salamon and Sokolowski’s re-conceptualization of the third sector. Voluntas.

[9] Salamon LM, Anheier HK (1992). In search of the non-profit sector. I: The question of definitions. Voluntas.

[10] Anheier HK. Nonprofit organizations: theory, management, policy: Routledge; 2005. http://books.google.com/books?id=s_uzg-KoVyIC&pgis=1

[11] Morris S. Defining the non-profit sector: some lessons from. History. 2000; http://eprints.lse.ac.uk/29032/1/cswp3.pdf. Accessed 29 Mar 2017

[12] Rees J, Mullins D, editors. The third sector delivering public services: developments, innovations and challenges. Bristol: Policy press; 2016.

[13] Barron DN, West E (2017). The quasi-market for adult residential care in the UK: do for-profit, not-for-profit or public sector residential care and nursing homes provide better quality care?. Soc Sci Med.

[14] Moore MH (2000). Managing for value: organizational strategy in for-profit, nonprofit, and governmental organizations. Nonprofit Volunt Sect Q.

[15] Dart R (2004). Being “business-like” in a nonprofit organization: a grounded and inductive typology. Nonprofit Volunt Sect Q.

[16] Hogg E, Baines S (2011). Changing responsibilities and roles of the voluntary and community sector in the welfare mix: a review. Soc Policy Soc.

[17] Kelly J (2007). Reforming public services in the UK: bringing in the third sector. Public Adm.

[18] Cabinet Office (2011). Open public services white paper.

[19] Office for Civil Society (2010). Building a stronger civil society.

[20] Kegeles SM, Rebchook G, Tebbetts S, Arnold E (2015). Facilitators and barriers to effective scale-up of an evidence-based multilevel HIV prevention intervention. Implement Sci.

[21] Chapman T, Bell V, Robinson F (2011). Measuring impact: easy to say, hard to do: a think-piece to stimulate sector debate from the Third Sector Trends Study.

[22] Gibbs L, Gambrill E (2002). Evidence-based practice: counterarguments to objections. Res Soc Work Pract.

[23] Sackett DL, Rosenberg WMC, Gray J a M, Haynes RB, Richardson WS (1996). Evidence based medicine: what it is and what it isn’t. Br Med J.

[24] Fraser MW, Galinsky MJ (2010). Steps in intervention research: designing and developing social programs. Res Soc Work Pract.

[25] Magill M (2006). The future of evidence in evidence-based practice: who will answer the call for clinical relevance?. J Soc Work.

[26] What Works? 2014. www.nice.org.uk. Accessed 29 May 2017.

[27] Project Oracle. http://project-oracle.com/.

[28] Blueprints for Healthy Child Development. http://www.blueprintsprograms.com/.

[29] Effective Interventions https://effectiveinterventions.cdc.gov/. Accessed 17 Nov 2017.

[30] Margaret Dolcini M, Gandelman AA, Vogan SA, Kong C, Leak TN, King AJ (2010). Translating HIV interventions into practice: community-based organizations’ experiences with the diffusion of effective behavioral interventions (DEBIs). Soc Sci Med.

[31] Amodeo M, Lundgren L, Cohen A, Rose D, Chassler D, Beltrame C (2011). Barriers to implementing evidence-based practices in addiction treatment programs: comparing staff reports on motivational interviewing, adolescent community reinforcement approach, assertive community treatment, and cognitive-behavioral therapy. Eval Program Plann..

[32] Ubbink DT, Guyatt GH, Vermeulen H (2013). Framework of policy recommendations for implementation of evidence-based practice: a systematic scoping review. BMJ Open.

[33] Gray M, Joy E, Plath D, Webb SA (2013). What supports and impedes evidence-based practice implementation? A survey of Australian social workers. Br J Soc Work.

[34] Montgomery P, Underhill K, Gardner F, Operario D, Mayo-Wilson E (2013). The Oxford implementation index: a new tool for incorporating implementation data into systematic reviews and meta-analyses. J Clin Epidemiol.

[35] Durlak JA, DuPre EP (2008). Implementation matters: a review of research on the influence of implementation on program outcomes and the factors affecting implementation. Am J Community Psychol.

[36] Hasson H (2010). Systematic evaluation of implementation fidelity of complex interventions in health and social care. Implement Sci.

[37] Durlak JA, Wells AM (1997). Primary prevention mental health programs for children and adolescents: a meta-analytic review. Am J Community Psychol.

[38] Glasziou P, Meats E, Heneghan C, Shepperd S (2008). What is missing from descriptions of treatment in trials and reviews?. BMJ Br Med J.

[39] Mihalic SF (2004). The importance of implementation fidelity. Emot Behav Disord youth.

[40] Basch CE, Sliepcevich EM, Gold RS, Duncan DF, Kolbe LJ (1985). Avoiding type III errors in health education program evaluations: a case study. Health Educ Q.

[41] Dobson D, Cook TJ (1980). Avoiding type III error in program evaluation. Eval Program Plann.

[42] Hasson H (2010). Systematic evaluation of implementation fidelity of complex interventions in health and social care.

[43] McCord J (2003). Cures that harm: unanticipated outcomes of crime prevention programs. Ann Am Acad Pol Soc Sci..

[44] Dishion TJ, Mccord J, Poulin F (1999). When interventions harm: peer groups and problem behavior. Am Psychol.

[45] Petrosino A, Turpin-Petrosino C, Hollis-PeelME, Lavenberg JG. ’Scared Straight’ and other juvenile awareness programs for preventing juvenile delinquency. Cochrane Database Syst Rev. 2013;(4):CD002796. 10.1002/14651858.CD002796.pub2. PMC11973542 23862186

[46] NCVO (2017). NCVO UK Civil Society Almanac.

[47] Lundgren L, Krull I, Zerden L de S, Mccarty D (2011). Community-based addiction treatment staff attitudes about the usefulness of evidence-based addiction treatment and CBO organizational linkages to research institutions. Eval Program Plann..

[48] Ramanadhan S, Crisostomo J, Alexander-Molloy J, Gandelman E, Grullon M, Lora V (2012). Perceptions of evidence-based programs among community-based organizations tackling health disparities: a qualitative study. Health Educ Res.

[49] Owczarzak J, Dickson-Gomez J (2011). Provider perspectives on evidence-based HIV prevention interventions: barriers and facilitators to implementation. AIDS Patient Care STDs.

[50] Martínez G, Sardiñas LM, Acosta-Perez E, Medina L, Rivera M, Pattatucci A. Capacity needs in community-based organizations for enhancing translational research in Puerto Rico. Prog Community Health Partnersh. 2014;8:53–60. https://search.proquest.com/docview/1529846240?accountid=13042. 10.1353/cpr.2014.0009 PMC4113094 24859102

[51] Lundgren L, Amodeo M, Cohen A, Chassler D, Horowitz A, Schoenwald S (2011). Modifications of evidence-based practices in community-based addiction treatment organizations: a qualitative research study. Addict Behav.

[52] Kimber M, Barwick M, Fearing G (2012). Becoming an evidence-based service provider: staff perceptions and experiences of organizational change. J Behav Health Serv Res.

[53] Mullen E, Shlonsky A (2005). From concept to implementation: challenges facing evidence-based social work. Evid Policy.

[54] Moher D, Liberati A, Tetzlaff J, Altman DG (2010). Preferred reporting items for systematic reviews and meta-analyses: the PRISMA statement. Int J Surg.

[55] Shamseer L, Moher D, Clarke M, Ghersi D, Liberati A, Petticrew M, et al. Preferred reporting items for systematic review and meta-analysis protocols (PRISMA-P) 2015: elaboration and explanation. BMJ. 2015;349 http://www.bmj.com/content/349/bmj.g7647. Accessed 16 Aug 2017 10.1136/bmj.g7647 25555855

[56] Ouzzani M, Hammady H, Fedorowicz Z, Elmagarmid A (2016). Rayyan—a web and mobile app for systematic reviews. Syst Rev..

[57] Thomas J, Harden A, Newman M (2012). Synthesis: combining results systematically and appropriately.

[58] Tricco AC, Cardoso R, Thomas SM, Motiwala S, Sullivan S, Kealey MR (2016). Barriers and facilitators to uptake of systematic reviews by policy makers and health care managers: a scoping review. Implement Sci.

[59] Gravel K, Légaré F, Graham ID (2006). Barriers and facilitators to implementing shared decision-making in clinical practice: a systematic review of health professionals’ perceptions. Implement Sci.

[60] Oliver K, Innvar S, Lorenc T, Woodman J, Thomas J (2014). A systematic review of barriers to and facilitators of the use of evidence by policymakers.( Research article). BMC Health Serv Res.

[61] Demby H, Gregory A, Broussard M, Dickherber J, Atkins S, Jenner LW (2014). Implementation lessons: the importance of assessing organizational “fit” and external factors when implementing evidence-based teen pregnancy prevention programs. J Adolesc Health.

[62] Rees R, Oliver K, Woodman J, Thomas J. Children’s views about obesity, body size, shape and weight: a systematic review. 2009. http://eppi.ioe.ac.uk/cms/Portals/0/Obesity%20Views%20Children%20R2009Rees.pdf?ver=2010-12-22-121209-040. Accessed 3 Apr 2017. 10.1186/1471-2458-11-188 PMC3072952 21439062

[63] Verboom B, Montgomery P, Bennett S. What factors affect evidence-informed policymaking in public health? Protocol for a systematic review of qualitative evidence using thematic synthesis. Syst Rev. 2016;5 10.1186/s13643-016-0240-6. 10.1186/s13643-016-0240-6 PMC4831125 27080993

[64] Maharaj R (2010). Organizational culture, absorptive capacity, and the change process: influences on the fidelity of implementation of integrated dual disorder treatment in community-based mental health organizations a.

[65] Pemberton GC. How are implementation and adaptation of evidence-based interventions applied in community practice settings? Lessons from the modelo de intervencion psicomedica. 2012. https://sph.unc.edu/files/2013/08/pemberton.pdf.

[66] Belza B, Petrescu-Prahova M, Kohn M, Miyawaki CE, Farren L, Kline G (2015). Adoption of evidence-based health promotion programs: perspectives of early adopters of Enhance®Fitness in YMCA-affiliated sites. Front Public Heal.

[67] Dippolito M, Lundgren L, Amodeo M, Beltrame C, Lim L, Chassler D (2015). Addiction treatment staff perceptions of training as a facilitator or barrier to implementing evidence-based practices: a national qualitative research study. Subst Abus.

[68] Flores R, Naranjo C, Hein A (2016). Use of evidence in the implementation of social programs: a qualitative study from Chile. J Evidence-Informed Soc Work..

[69] Gandelman A, Dolcini MM (2012). The influence of social determinants on evidence-based behavioral interventions-considerations for implementation in community settings. Transl Behav Med.

[70] Lattimore D, Griffin SF, Wilcox S, Rheaume C, Dowdy DM, Leviton LC (2010). Understanding the challenges encountered and adaptations made by community organizations in translation of evidence-based behavior change physical activity interventions: a qualitative study. Am J Health Promot.

[71] Owczarzak J (2012). Evidence-based HIV prevention in community settings: provider perspectives on evidence and effectiveness. Crit Public Health.

[72] Payan DD, Sloane DC, Illum J, Vargas RB, Lee D, Galloway-Gilliam L (2017). Catalyzing implementation of evidence-based interventions in safety net settings: a clinical-community partnership in South Los Angeles. Health Promot Pract.

[73] Petrescu-Prahova M, Belza B, Kohn M, Miyawaki C (2016). Implementation and maintenance of a community-based older adult physical activity program. Gerontologist.

[74] Pinto BM, Waldemore M, Rosen R (2015). A community-based partnership to promote exercise among cancer survivors: lessons learned. Int J Behav Med.

[75] Veniegas RC, Kao UH, Rosales R (2009). Adapting HIV prevention evidence-based interventions in practice settings: an interview study. Implement Sci.

[76] Collins CB, Hearn KD, Whittier DN, Freeman A, Stallworth JD, Phields M (2010). Implementing packaged HIV-prevention interventions for HIV-positive individuals: considerations for clinic-based and community-based interventions. Public Health Rep.

[77] Murray M, Culver T, Farmer B, Jackson LA, Rixon B. From theory to practice: one agency’s experience with implementing an Evidence-Based Model Maureen. J Child Fam Stud. 2014;5:844–53. 10.1007/s10826-013-9738-x PMC4167773 25242876

[78] Feutz K, Andresen P (2013). Cuidate: implementation of a culturally based sexual risk reduction program for Hispanic adolescents. Hisp Heal Care Int.

[79] Honeycutt S, Carvalho M, Glanz K, Daniel SD, Kegler MC (2012). Research to reality. J Public Heal Manag Pract.

[80] House LD, Tevendale HD, Martinez-Garcia G (2017). Implementing evidence-based teen pregnancy-prevention interventions in a community-wide initiative: building capacity and reaching youth. J Adolesc Health.

[81] Lundgren L, Chassler D, Amodeo M, D’Ippolito M, Sullivan L (2012). Barriers to implementation of evidence-based addiction treatment: a national study. J Subst Abus Treat.

[82] Thomas R, Zimmer-Gembeck MJ, Chaffin M (2014). Practitioners’ views and use of evidence-based treatment: positive attitudes but missed opportunities in children’s services. Adm Policy Ment Heal Ment Heal Serv Res..

[83] Hunter SB, Han B, Slaughter ME, Godley SH, Garner BR (2017). Predicting evidence-based treatment sustainment: results from a longitudinal study of the adolescent-community reinforcement approach. Implement Sci.

[84] Allicock M, Johnson L-S, Leone L, Carr C, Walsh J, Ni A (2013). Promoting fruit and vegetable consumption among members of black churches, Michigan and North Carolina, 2008–2010. Prev Chronic Dis.

[85] Vanderpool R, Gainor S, Conn M, Spencer C, Allen A, Kennedy S (2011). Rural remote health. Rural Remote Health.

[86] Kegeles SM, Rebchook G, Pollack L, Huebner D, Tebbetts S, Hamiga J (2012). An intervention to help community-based organizations implement an evidence-based HIV prevention intervention: the Mpowerment project technology exchange system. Am J Community Psychol.

[87] Macmillan R, Ellis A, With P, Kara H, Dayson C, Sanderson E (2014). Building capabilities in the voluntary sector: what the evidence tells us. TSRC Research Report 125.

[88] Building capacity: Research - Big Lottery Fund. https://www.biglotteryfund.org.uk/research/making-the-most-of-funding/building-capacity. Accessed 14 Dec 2017.

[89] Collins C, Harshbarger C, Sawyer R, Hamdallah M (2006). The diffusion of effective behavioral interventions project: development, implementation, and lessons learned. AIDS Educ Prev.

[90] Collins CB, Sapiano TN (2016). Lessons learned from dissemination of evidence-based interventions for HIV prevention. Am J Prev Med.

[91] Peterson AS, Randall LM (2006). Utilizing multilevel partnerships to build the capacity of community-based organizations to implement effective HIV prevention interventions in Michigan. AIDS Educ Prev.

[92] Michie S, Fixsen D, Grimshaw JM, Eccles MP (2009). Specifying and reporting complex behaviour change interventions: the need for a scientific method. Implement Sci.

[93] Gearing RE, El-Bassel N, Ghesquiere A, Baldwin S, Gillies J, Ngeow E (2011). Major ingredients of fidelity: a review and scientific guide to improving quality of intervention research implementation. Clin Psychol Rev.

[94] Weersing VR, Rozenman M, Gonzalez A (2009). Core components of therapy in youth. Behav Modif.

[95] Maggin DM, Johnson AH (2015). The reporting of core program components: an overlooked barrier for moving research into practice. Prev Sch Fail Altern Educ Child Youth.

[96] Hoffmann TC, Glasziou PP, Boutron I, Milne R, Perera R, Moher D (2014). Better reporting of interventions: template for intervention description and replication (TIDieR) checklist and guide. BMJ.

[97] Hawe AT, P Shiell R (2004). Complex interventions: how “out of control” can a randomised controlled trial be?. BMJ.

[98] Michie S, van Stralen MM, West R (2011). The behaviour change wheel: a new method for characterising and designing behaviour change interventions.

[99] Légaré F, Ratté S, Gravel K, Graham ID (2008). Barriers and facilitators to implementing shared decision-making in clinical practice: update of a systematic review of health professionals’ perceptions. Patient Educ Couns.

[100] Mitchell GE, Berlan D (2016). Evaluation and evaluative rigor in the nonprofit sector. Nonprofit Manag Leadersh.

[101] Harlock J (2013). Impact measurement practice in the UK third sector: a review of emerging evidence. TSRC Working Paper 106.

[102] Arvidson M (2014). Evidence and transparency in the open public services reform: perspectives for the third sector. TSRC Working Paper 117.

[103] Carmel E, Harlock JE (2008). Instituting the “third sector” as a governable terrain: partnership, procurement and performance in the UK. Policy Polit.

[104] Maier F, Meyer M, Steinbereithner M (2016). Nonprofit organizations becoming business-like: a systematic review. Nonprofit Volunt Sect Q.

[105] Moher D, Schulz KF, Simera I, Altman DG (2010). Guidance for developers of health research reporting guidelines. PLoS Med.

[106] Hsu C, Sandford B (2007). The delphi technique: making sense of consensus. Pract Assessment, Res Eval.

[107] Thomas J, Harden A. Methods for the thematic synthesis of qualitative research in systematic reviews. BMC Med Res Methodol. 2008;8(45) 10.1186/1471-2288-8-45. 10.1186/1471-2288-8-45 PMC2478656 18616818

[108] Bach-Mortensen AM, Montgomery P (2018). What are the barriers and facilitators for third sector organisations (non-profits) to evaluate their services? A systematic review. Syst Rev..

[109] Sackett D. Evidence-based medicine: how to practice and teach EBM. Churchill Livingstone. 2000; 10.1016/S1031-170X(97)80036-0.

[110] Chalmers I (2005). If evidence-informed policy works in practice, does it matter if it doesn’t work in theory?. Evid Policy..

[111] Chalmers I (2003). Trying to do more good than harm in policy and practice: the role of rigorous, transparent, up-to-date evaluations. Ann Am Acad Pol Soc Sci.

[112] WHO. Deworming in children: WHO; 2018. http://www.who.int/elena/titles/deworming/en/. Accessed 22 Apr 2018

[113] Miguel E, Kremer M (2004). Worms: identifying impacts on education and health in the presence of treatment externalities. Econometrica.

[114] Welch VA, Ghogomu E, Hossain A, Awasthi S, Qar Z, Bhutta A (2017). Mass deworming to improve developmental health and wellbeing of children in low-income and middle-income countries: a systematic review and network meta-analysis.

[115] Taylor-Robinson DC, Maayan N, Soares-Weiser K, Donegan S, Garner P, Taylor-Robinson DC (2015). Deworming drugs for soil-transmitted intestinal worms in children: effects on nutritional indicators, haemoglobin, and school performance. Cochrane Database of Systematic Reviews.

[116] McHugh RK, Barlow DH. The dissemination and implementation of evidence-based psychological treatments. A review of current efforts. Am Psychol. 2010;65:73–84. 10.1037/a0018121 20141263

[117] Proctor EK, Landsverk J, Aarons G, Chambers D, Glisson C, Mittman B (2009). Implementation research in mental health services: an emerging science with conceptual, methodological, and training challenges. Adm Policy Ment Heal Ment Heal Serv Res..

[118] Groene O, Botje D, Suñol R, Lopez MA, Wagner C (2013). A systematic review of instruments that assess the implementation of hospital quality management systems. Int J Qual Heal Care.

[119] Proctor E, Silmere H, Raghavan R, Hovmand P, Aarons G, Bunger A (2011). Outcomes for implementation research: conceptual distinctions, measurement challenges, and research agenda. Adm Policy Ment Heal Ment Heal Serv Res.

