# Supplementary material for: Barriers and facilitators to implementing evidence-based interventions among third sector organisations: a systematic review
Source: Implement Sci. 2018 Jul 30;13:103. doi: 10.1186/s13012-018-0789-7 (PMC6065156; doi:10.1186/s13012-018-0789-7)
Supplement: Supplementary file 2 — Contains the search strategy employed by the study. (PDF 51 kb) [file 13012_2018_789_MOESM2_ESM.pdf]

Search strategy via ProQuest	
**Databases:** ABI/INFORM Global, Applied Social Sciences Index & Abstracts (ASSIA), International Bibliography of the Social Sciences (IBSS), MEDLINE®, PAIS Index, Policy File Index, Social Services Abstracts, Sociological Abstracts, Worldwide Political Science Abstracts	
#1	ALL("evidence based practice*" OR "evidence-based practice*" OR "evidence-based intervention*" OR "evidence based intervention*" OR EBI* OR EBP* OR "evidence-based program*" OR "evidence based program* OR EBM OR "evidence-based service* OR "evidence based service*" OR evidence-based OR "evidence based")
#2	ALL(experience* OR attitude* OR perception* OR learning OR barrier* OR facilitat* OR challenge* OR benefit* OR success* OR constrain* OR difficult* OR enhanc* OR influen* OR interfer* OR motivat* OR obstruct* OR problem* OR promot* OR restrain* OR restrict* OR disincentive* OR factor* OR capacity OR enabler*)
#3	ALL(implement* OR adapt* OR adopt* OR adher* OR deliver* OR becom*)
#4	ALL(charity OR charities OR "charitable organisation*" OR "charitable organization*" OR "voluntary sector" OR "voluntary organisation*" OR "voluntary organization*" OR "third sector" OR third-sector OR "community organisation*" OR "community organization*" OR "community-based organisation*" OR "community-based organization*" OR non-profit* OR "not for profit" OR nonprofit* OR non-profit* OR "social enterprise*" OR TSO* OR CBO* OR VCO* OR VCS* OR "civic sector" OR "civil sector " OR "social sector" OR CSO* OR "non government*" OR NGO* OR NPO* OR "civil society" OR community-based OR "community based")
#5	1 AND 2 AND 3 AND 4
