# Supplementary material for: Barriers and facilitators to implementing evidence-based interventions among third sector organisations: a systematic review
Source: Implement Sci. 2018 Jul 30;13:103. doi: 10.1186/s13012-018-0789-7 (PMC6065156; doi:10.1186/s13012-018-0789-7)
Supplement: Supplementary file 4 — Contains the appendices referred to throughout the study. (DOCX 124 kb) [file 13012_2018_789_MOESM4_ESM.docx]

**Appendices**

**Appendix S1: Extracted quotes on barriers**

**Allicock et al. 2012**

No quotes on barriers were presented in this article.

**Amodeo et al. 2011**

Certification Process

- “burdensome and time consuming”
- “high certification requirements,’’
- “certification was difficult—constant fidelity checking”
- “having to record sessions creates rapport problems with clients.”

Funding

- ‘‘not enough funding to implement fully”

Client Resistance/Client Non-Engagement/Lack of Suitability

- “client resistance to participation”
- “lack of participation from caregivers”
- “It takes a lot of follow up to get . . .adolescents to stay engaged, and sometimes it also means more follow up with the parents as well. . .so it is a challenge for those who are really not married to the program”
- “concrete thinking—CBT more appropriate for people who have insightful thinking”
- “clients could not stay focused because of outside relationships with relatives/friends”
- “in CBT groups, clients who miss the first week of the program when CBT is explained and goals are set.”

Training

- “some staff without enough training are confused about the model”
- and [with so much staff turnover], ‘‘a barrier is keeping staff trained,”
- “it is easy for staff to ‘‘slip’’ from the MI framework; it requires constant clinical supervision; and a lot of time is required for appropriate clinical supervision.”

Philosophical Differences

- “There is a perceived conflict between MI and the 12-step approach”
- “the transition to MI from the previous theoretical orientation is difficult for some clinicians due to different backgrounds”

Rigidity of the Model

- “little room for diversity in procedures”
- “very few cultural adaptations”
- “the developers had a very difficult time seeing beyond this one particular model, which was very specific. . .so there’s absolutely no room to bring other orientations in”
- “clinician doesn’t have flexibility for home visits.”

Lack of Concrete Services

- “for homeless population, lack of housing is a barrier”
- “for co-occurring psychiatric and physical disorders, very limited resources”
- “lack of resources in county (no bus system, few jobs)”
- “project had limited access to substance abuse treatment.”

**August et al. 2006**

No quotes on barriers were presented in this article.

**Belza et al. 2014**

Competing programming

- “For group classes we have dance, water aerobics, step aerobics, spinning, and the range of movement class from [another exercise program]. We have additional programs that are available at a cost, and those include our nutritional services; the EF classes; swimming lessons; different sports programs, and then small group training types of classes” (Age 30,11 years with the Y).
- “We acknowledge that space is an issue… They [wellness directors] see it as oh we already have [another program], our program for active older adults. Why would we want to do this one?” (Age 38, 8 years with the Y).
- “We [offer EF] off-site. We are not in our own building anymore. It was to save on rent…The big room is often taken up with children’s camps and things like that” (Age 60, 7years with Y-affiliated site).
- “we no longer had the luxury of having two senior programs running because of space limitations.”
- “we were having a time and space crunch. It wasn’t anything wrong with the program *perse* but we’re not going to take away our already very strong programs and try something new.”

Limited resources and expertise

- “The staff did not see the benefit or the value to their people” (Age 45, 10years).
- “And getting our health and wellness directors to understand and not condemn it, like “What’s in it for us?” (Age 38,8 years with the Y).
- “Where are we going to put it; who is the instructor going to be; who’s going to pay for this, or where are the funds coming from” (Age 51, 16 years with the Y).
- “I know the whole issue is that people don’t have time. There is a lack of staff. We have it here, too, and so I know some of the issues (Age 65, 6 years with Y-affiliated site).

**Collins et al. 2010**

No quotes on barriers were presented in this article.

**D'Ippolito et al. 2015**

Characteristics of Training

- “Had an emphasis on training from book learning rather than experiential learning”
- “There has been no formal training yet and there are inconsistencies in training as not everyone was trained the same way”
- “Training was not that good.”
- “Some who go through training do not follow through, thinking it is too intense for them”
- “Training was five days and required travel”
- “Training requirements seemed unreasonable. Too much of staff’s time.”

Amount and Timing of Training

- “Peers weren’t trained to work on the services provided through this contract, so the model wasn’t really implemented”
- “Shelter staff weren’t originally trained in MI.”
- “Two years into operating with clients, training with MET and actual skill practice are very difficult. Skill building, even training once a month is not enough”
- “Additional trainings would have been worth it—not enough training.”
- “Lack of continual education on the model”
- “Ongoing consistent training and the time to be able to do that versus I got my MSW and therefore I am a CBT [practitioner].”
- “Not having enough time at the beginning of the grant to train because we have to hit the ground running”
- “Lack of training at the beginning.”
- “The amount of training that is needed to develop comfort with the model.”

Agency Capacity for Training

- “Finding the time to get staff trained because it takes them away from their work.”
- “Not enough money in grant for training or hiring master level clinicians.”
- “The overnight and residential staff were hard to pull away to trainings.”

Access to and Availability of Training

- “External trainings were far and few between, so when staff wanted training it was not always available.”
- “Costs are outrageous for trainings and certifications, $45,000.”
- “Getting to LA for the training so it could be taught to fidelity.”

Training Related to Specific Staff Needs

- “A quarter of the staff did not have enough continuing education and struggled with the new model”
- “Not enough people were trained. We need agency wide trainings”
- “The case managers couldn’t go to trainings, only the therapists.”
- “Providing excessive trainings due to turnover”
- “Keeping staff up to date with what [EBP] means.”
- “There are different education levels for our staff. It is diverse, so difficult to train all at once. They have different skill sets.”

**Demby et al. 2014**

No quotes on barriers were presented in this article.

**Dolcini et al. 2012**

Assess

- Organizational capacity
  - “So it was more a matter of elimination as opposed to “YES, we know how to do this stuff.” Because, in fact, it represented a totally new way of doing things for us and for the agencies we’re working with, and it probably wasn’t the best match. (ED)”
  - “I don’t want to be so crass as if it was just a process of elimination, but in a way we looked at all the things they [CDC] were going to fund, and we said, OK, what can we not do, and crossed those off right away. Then there were a few that were left . . . And, then we had a dialogue in relation to our organizational capacity was as far as what made sense, and we ended up with [intervention]. (ED)”
  - “We were part of a [name] demonstration project. And, that was coming to an end, so we were actively looking to how to not lay people off. (ED)”
  - “And it seemed like our only option was to do it in this format, which was through, applying through the DEBIs. . . . Because previously we had been doing much more of a traditional outreach model. And, not done a structured program. (ED)”

Select

- Attending all trainings (getting everyone up to speed)
  - “And it was a while before the training, not everybody went to trainings right away, so it took a series of months for everybody at all the sites to actually go through all the trainings. And only once you go through trainings did we understand, did they really understand what this intervention was, and what we were gonna be expected to do. (ED)”

Prepare

- Staff turnover
  - “Well, there wasn’t a lot of structural changes…. It was more hiring new staff…I think for us it was the challenge of starting up a new intervention. (ED)”
  - “We were in the process of implementation, within the first two months of finally getting our feet wet and starting our groups, one of the individuals resigned and moved…. so we were kind of hindered a bit…The next outreach worker also left our agency…It took us a long time to get a replacement. (ED)”

Implementation

- Clash between research and reality
  - “And so we feel very limited because … this is the enrollment session for the program, and so there’s just this clash of, it’s like a culture clash to me around the culture of research and the culture of the street. And the reality of these people’s lives. So, it just doesn’t seem like a very successful way to begin that relationship with them. (ED)”
- New to Staff
  - “Um, it was kind of a process of trial and error of how, um, a lot of the, a lot of pieces of the intervention were new to us and new to the staff. Especially like for the social…. What technique works best when introducing this to the client, on inviting people and friends in. It took us a while to kind of figure that one out and find something that was effective. (ED)”
- Monitoring and Evaluation
  - “We’re evaluating in the sense of the basic three forms that we have. We have a pre and a post test and [a specific form that another agency requires]. Those are the three forms that I know of that are components of it [evaluation]…The concern, the question that we have is, How do you evaluate the data we provide? What does it mean? And, we don’t have a system in place for that [evaluation]. (I)”
  - “…I know the evaluation form, but I sometimes don’t believe in this form. Because the people don’t say all… When, after the session, we talk to the clients, we ask them how they feel, what they think about the session. Most clients say that it’s good, it’s helpful. Most of the clients disclose their sexual orientation, their HIV status in the process. But this is not the reason [I think it is successful]. The reason is lower stress. Some people told us, “I feel good, no more stress.” Maybe they don’t or won’t disclose their status, but they don’t have stress anymore. And, it’s good. This is the reason that this intervention is working. (I) (Although defining stress and improving coping skills are core elements of this DEBI, it is not a formal outcome for the intervention.)
  - “…I think there are probably [some behavior changes]. It’s just a guess. I can’t say that the intervention is effective. I can’t say that it’s not. (I)”

**Feutz et al. 2013**

No quotes on barriers were presented in this article.

**Flores et al. 2016**

Programs do not use their existing processes

- “The methodology is determined from the state agency . . . everything is determined by the agency, sometimes you must visit people, the hours are for training, the methodology to be used, instruments, all is defined by the agency.”
- “We cannot recollect data. Database comes from the agency. All we ask users is to complete on the computer platform of the agency. The information is from the agency.”

Lack of formal training in EBP – rely on literature

- “That’s the beauty of this foundation. We work under the model of systems theory. Also we are reviewing ecological models. We try to be always updated on theoretical frameworks, as the situation of young people and children is changing through time. The phenomenon involved is not static, certain types of social vulnerabilities are increasing.”
- “As you grow professionally you’re using more complex frameworks. You start your bibliography from your university world searches, social workers look at the theory of systems, then all our frameworks come from there. Today it is more complex, because the agency is increasingly stronger in the development of conceptual frameworks.”

Unable to appraise evidence

- “As the evidence is in Spanish, the language is important for decision making.”
- “I think the biggest obstacle has to do with language, we are not able to read any language.”

Accessibility of EBP information

- “That literature is easily accessible, with updated information.”

Time constraints

- “I think the issue of lack of time is a barrier. The times in which we work are also barriers. The lack of time in which we organize the work does not give the possibility to read literature, attend seminars, and do more and more research.”

Uninvolved in design

- “We are just executors, we implemented a project that is methodologically designed and we only indicate the timing and build the team that is in charge of the execution.”
- “That is information that I do not drive, because all this comes from the agency, which has a department of study and they have seen international experiences. I do not know what other actors they have consulted.”
- “The object of intervention in this program is defined by the agency, it is defined based on how this project will work. We have no interference in defining the target population. And this is defined by the agency. We only select the population based on the requirements defined by the agency”

**Gandelman et al. 2012**

Language

- “Well, the first change was to change the language, to translate the curriculum. . . . So all the curriculum had to be translated.”
- “…one particular challenge that we're dealing with is the enrollment form. You have to ask them 15 questions which have six parts to each question roughly....So it's more of a clash between the research and the practical. We have a population that's um, hesitant to give out personal information, who if it seems anything formal or government driven or whatever, they're just very suspicious and unlikely to respond....our work…is based on building rapport and having trust,…relationship building is THE most important thing and right now we're coming in and having an interview, a formal research interview at the beginning.”

Identity issues: sexual and ethnic identity

- “…class had to do with even the conversations they wanted to have…regarding relationships....The folks with the higher socioeconomic backgrounds were having different discussions around dating versus folks who were at the lower spectrum that might have been doing things for money....”
- “…if you have a 20-year-old sitting with a 40-yearold, it's very different, and we have class differences. We do have people who identify as gay or bi, and some who do not identify…and that's a challenge in having two different groups of individuals in the room. And we also have people who are not a person of color and would say they are…and they're really interested in the material and that's really hard.”

Stigma

- “…because to be realistic, most of these clients [at our agency] do sex work for a living....But for them to accept that they're HIV positive, and the potential ramifications that it might have, legal and economic....Because it's a small community.... so we have to open up a completely different day for them to feel safe....we have a more challenging time bringing them into this intervention because of that factor, their sex work. So, it's stigma about being HIV positive (and their ongoing sex work).”

Legal status and incarceration

- “…we pride ourselves on our efforts to keep confidentiality a priority, so it's a hurdle as far as advertising....it's kind of been a struggle that way, because we know they're out there but finding them in the best manner is what's hard.”
- “They have to move around quite often. They have been pressured by the police. Some of them, specially the Latino population, they don't have documents to be in this country, so…[they]have to move and they are more persecuted than any other population. Also this population that you're seeing today, tomorrow you don't.”

Drug use and homelessness

- “…this population is SO challenging…I don't really understand how other sites or how the original research group was able to get people to come in and come in again and come in again…the same group....it's like the most unstable population. These are people who, you see them in the street, you tell them about it, they say, yeah I'll come. You see them an hour before, they say yeah, I'll come, 15 min maybe you see them; we've actually…sent people out like the hour before to round people up, and they don't show up.

**House, et al. 2017**

No quotes on barriers were presented in this article.

**Hunter et al. 2017**

No quotes on barriers were presented in this article.

**Honeycutt et al. 2012**

Financial constraints

- “They thought that if we had some type of [party or family] event, [employees] would be saying, “Now you’re asking me to take unemployment weeks, but . . . you’re having an event.” (Site coordinator)”

**Kegeles et al. 2015**

HIV prevention system factors

- The entire HIV prevention system affects intervention implementation
  - Funders
    - “When he [the coordinator] met with XXX, the guy from the health department…they don’t want to pay for him to do activities that are associated with social events [a major Core Element of the intervention]…they only want to pay him to do the M-groups and the outreach.”
- Knowledge about intervention
  - “The biggest difference between the way they do the model is that they have to follow the contract that the county lays out, which takes it far from the intervention…they have to do tons of things that are different from the model.”
- Belief if efficacy of intervention
  - “They [the core group] don’t plan anything as a group…the curriculum is ideal but unrealistic for real life as they know it…people are busy… so the coordinators do the events…she thinks that that part of the program, the core group planning stuff, is a joke and needs to be changed…maybe in a place where there is nothing to do but pick your nose, but in [a large city], it isn’t going to happen.”
- Desire to change agency’s existing approach
  - “They had been trying to get it [MP] going…or something like it….they had a lot of people coming, but it wasn’t focused…it didn’t look like the model…the agency didn’t really care. They do outreach once a week at the clubs, and they do referrals for [HIV] testing and STD testing… they go through and give out safer sex kits to anyone who will take them from them....[but] they haven’t redeveloped the kits for Mpowerment.”
- Accountability for work
  - “When she [the supervisor] goes to talk to [the ED] and she tells him about stuff that is going on with the project, he just sits there and nods…he doesn’t provide any direction for her and is more apt to err on the side of supporting the coordinators rather than questioning if they are a good fit or not…this makes it difficult for her…when she thinks that they may need to be fired.”
- Appropriateness and capacity of individuals for coordinator positions
  - “She hired someone on recently who has a marketing background… who is tuned in with what is attractive and fun… but when it comes to promoting something…he is like ‘I don’t know, I don’t know…I don’t know’…she thinks that even with the training, and with the weekly meetings…he feels a bit overwhelmed…he is good at databases and stuff like that…but publicity, marketing, recruitment…it is very hard.”
- Organizational stability
  - “He [the funder] thinks that the project needs to go to a different organization…the agency isn’t stable enough.”

Community factors

- Geography
  - “Trying to compete with San Francisco [to attract YGBM] wasn’t working” [respondent’s CBO was located in a county only a few miles away]
- Sociopolitical context
  - “Publicity was hard because they couldn’t even have a website that was geared toward gay men because the county is so conservative and the funder [the county health department] didn’t want to risk creating a commotion.”

Intervention factors

- Intervention characteristics
  - “When he went to Atlanta [to a conference], he saw [a presenter connected with the MP research] and he talked to him and went to his presentation, and at the time they didn’t like the [intervention] because they thought that they [the researchers] had all this money to do it and it wasn’t the real world…because they were talking to CBOs that were working on shoestring budgets and they don’t have lots of money for training and planning like they [the researchers] had.”
- Adaptation issues
  - “The question of fidelity is something that they talked about a lot… the boxed [DEBI] interventions are great, but what people really need is more TA about how to effectively adapt these interventions while retaining the theoretical core. They [the agency] needs to build their capacity to understand the internal logic of the M-group piece of the intervention so that they can say ‘here is the logic of this activity, and the behavior it is seeking to address…here is our target population for this intervention…how do we change M-groups for this target population while retaining fidelity to the original design?’”

**Kimber et al. 2012**

Having clear leadership

- “Flounder”
- “Run around in circles”
- “Need to request extended deadlines and support in completing tasks.”

Supplying needed resources

- “Like when I was starting on that working group, I was also meeting [the demands of] our accreditation process. So, you know, I kind of had to find the balance between doing both of those things, knowing that the one—the accreditation, had a specific timeline. So, I said to the group, you know, we are about to do this (accreditation), and if you want to go on to the next steps, it will have to wait until another time, because these other things are up.”

Doing this differently

- “My issue is that some of the trainings are not even provided by our own staff. Yes, we need professional training… but using our own resources, like we have done for some of the Triple P training, and if we could do that for some of the other EBP trainings that will be rolled out, that would be good.”
- “There are still people in the agency that are involved in this (CT) that shouldn’t be, because they don’t get it, and they don’t try. It is clear that this isn’t their priority; it’s more just on their plate. Because there are some people that do this really well, and then there are others who struggle, and then the strugglers keep coming, saying I can’t get it, I can’t get it. Well, that’s because you can’t do it.”

**Lattimore et al. 2010**

Program logistics

- Readability of material ("small fonts, literacy level above the recommended fifth- to sixth-grade reading level, or appropriateness of graphics, pictures, and examples")
  - ‘‘The example in the book or on the website doesn’t include retired people; therefore, their situations are not similar. The examples don’t look like me and they have different lifestyles….’’

Program theory

- Tracking thoughts related to physical activity
  - “Participants are not completing the ‘keeping track of thoughts’ and ‘keeping track of PA.’ They [participants] all commented that they do think about PA all the time, but the tool to track it isn’t simple or friendly.’’

Program philosophy

- Adhering to screening and eligibility requirements
  - ‘‘Moderate intensity is like you are rushing to get to the bus before it pull away.’’
  - “The functional level is so low for some participants that if they can consistently engage in activity over the course of the program, that is huge.’’
- Assessing light versus moderate activity
  - “Everyone has a different perspective of what light activity is versus moderate activity.’’

**Lundgren et al. 2011**

Lack of fit with current organizational practice

- “The other thing that we, we had to do is this is meant to be a closed group, in other words you have your women, they always come to the same group, it's always the same women. And that was not doable for us at all. So we had to adapt it…to be an open group, and again…one did not have the sense that they [the funder]were happy about any of this for us. So…I don't know that we have a whole lot of fidelity in the pure sense going on here, although I don't think we changed any of the curriculum or anything.”

Lack of fit with organization’s population

- “…in this program we don't do the full [EBP] because it's with [client population], and we had to tone it down a little bit, cause the true [EBP] is just too aggressive. It doesn't really work with them….”
- “You know we have diverse populations and so some of the – a couple – there's seven different types of ethnic populations that we're serving. Three of them I think the [EBP] fits very well. The other four populations, we have to do a lot of modifications. So that's why when you say – you're asking me the scales – it's actually a scale for all seven. But if for three of them we didn't need to modify very much, maybe at a two or a three level, but the others are more a six or seven level.”
- “We have over the past years, because…predominantly our population is [client population], and…so we've made adaptations to…how you move people through [EBP]. That looks different culturally.”
- “We did…they'll tell ya…with the [EBP] about the four legs of the stool…and all the different components that need to be there, and [client population] is one that when we talked to the folks they said ‘we're not even sure if this…can completely work with [client population] because of the lack of supports that they have in their lives.’”
- “It's really kind of ingrained in…‘if that's not working then try something else.’ I know we have done a lot of the case management while at the same time doing the[EBP], so it's not really [EBP] not standing alone, but…[specific client population] have so many different needs…that we try and support them when we can, and that's why we…add some of the case management alongside the [EBP].”

**Lundgren et al. 2012**

No quotes on barriers were presented in this article.

**Marahaj 2010**

Philosophical orientation

- Level of congruence
  - “Program Philosophy Score (3): There continues to be a basic IDDT orientation articulated by all sources interviewed, however elements of the model that remain problematic, e.g. lack of group DD treatment, provision of substance abuse treatment outside the agency, and generic client goals/plans, have not progressed significantly, despite their centrality to the EBP's core principles. Although consumer and family interviews had been scheduled, the interviews did not take place. An agency brochure that communicates the IDDT philosophy has not yet been developed. Some stakeholders from other systems who refer clients to the agency (and elsewhere) do not yet exhibit a good understanding of the model's core components. (LF1, 24M Fidelity Report) Program Philosophy, score was 2. In speaking with staff, at various levels of the organization, a focus appears on getting to the destination of IDDT. Helping staff see this program in a long-term, if not permanent, manner, will likely help LF3 to more fully implement IDDT. Identifying ways for the team to communicate this to one another and to reviewers will assist this organization with fidelity. (LF3, 12M Fidelity Report)”
- Norms, values, and beliefs
  - “Rather than outreach, motivational interventions, and other engagement techniques for people in the engagement and motivational stages of recovery, the team members seemed to talk more about setting boundaries for clients by not helping them find new housing when they lose old housing due to drug use. (LF2, Baseline, Fidelity Report)”
  - “They are pretty down on this implementation as well. They see it as a barrier to their own productivity. They see the training as long and useless, and they really don't think this program has been very helpful to them. They don't see clients in the stage of change the way that IDDT promotes. They don't really see much hope for many of these clients. (LF3, 18M Implementation Monitor Summary)”
  - "Some team members were uncomfortable with IDDT's support for attempts to reduce rather than immediately eliminate substance use."
  - "There are however, a few resistant team members, according to the program leader, who are not comfortable with letting clients make their own choice.” This attitude was highlighted in the 6M consultant/trainer’s interview for LF3: "Their attitudinal set going into this was very paternalistic and controlling," in addition to "they don't really value consumers input all that much," while "some staff talked about clients in a non-professional and negative way.”
  - “That is the ongoing issue that this team has been dealing with since the beginning of this project. Trying to figure out where to put their IDDT team has been a huge issue for this team. I believe I read this is the largest mental health service agency in the state, so perhaps it’s that bureaucracy that is preventing this organization from finding the proper place for its IDDT team. (LF2, 12M Implementation Monitor Summary)”
  - We've talked all along in the implementation process that if you anchor the program in a person or persons it's not going to fly. You have to anchor in the culture and they're one site where it's not anchored in the culture (HF1, 6M Trainer Interview)

Leadership

- Role of leadership
  - “I think there is definitely a misconception from program leaders. They are a little out of touch with what their staff does on a daily basis.”
  - “I think the program leader has not been extremely effective...he speaks very passively about he is just doing what his administrators tell him to do; he is not invested in the model. He doesn’t care about the model. He doesn’t particularly believe that the model will work better than what he is already doing and he disagrees with us in terms of how closely they adhere to the model (LF3, 6M Implementation Monitor Summary)”
  - ““The lack of clarity around this important part of the multi-disciplinary team is symptomatic of the way the program came to be structured.”
  - “Agency administration was astute in recognizing the value of IDDT over parallel services but underestimated the complexity of the model and what it would take to implement it. One way that this underestimation played out was the assignment of IDDT program roles to staff who were either not “with the program” or not adequately skilled to carry out those roles. (LF1, 6M Implementation Monitor Summary)”
  - “She was just describing those behavioral acts as things they were motivational counseling. So, I don’t really think she understands what it is. It is a very important part of stage wise interventions, it is a very important element of outreach and engagement but I just don’t think she understands what it is so she is not able to lead her staff and tell them what motivational counseling is. (LF2, 6M Implementation Monitor Summary)”
  - “As if the Program Leader or Registered Nurse went to Borders, bought a book on “here’s this great new thing to do with your group” and just start doing it unbeknownst to the rest of the agency and that is the way it will probably happen and if somebody said you can interview the upper administration a year from now and say “so how is the…work you’re doing here?” and they can say “what’s that?” (HF1, 6M Program Leader Interview)”
  - “About [the CEO], ….in spite of whatever he brings to the table personality wise, he got that agency out of the red and into the black and his fiscal management was a clear strength. If you got underneath the questions, they were fiscal questions and that over time the fiscal viability of the team has been demonstrated to him, he's now sort of with the program. …….when he gets up there endorsing it, he's not endorsing fidelity, he's endorsing the viability of it, the outcomes and the fiscal viability (HF1, 24M Trainer Interview)”
- External leadership
  - “Steering committee acts as steering committee for both the ACT and IDDT implementations. I suspect that having a steering committee overseeing both practices might be diluting the steering committee’s mission too much.”
  - “The former implementation monitor has noted that the consumer and family member involvement at this agency is very minimal. For example, she said that the agency picked a consumer and a family member who would be nonassertive for this steering committee.”

Adaptability

- Consensus and buy-in
  - “I think another one is just kind of well, "this whole intensive thing is just a little bit much for me, I kind of like the idea of a 40-1 deal where it's more generic and I don't have somebody that wants to kill themselves every week," and so I think there are some people who are hanging in on the team because they're good people and they're team players but they're not particularly invested in where they are. (LF1, 6M Program Leader Interview)”
  - “...So there is a range in attitude toward the model with some people being somewhat enthusiastic about it all the way down to people where you can just tell they are rolling their eyes and they don’t want to be involved. It is just a real mix of how they can really get this model going. I asked a couple of people why they think they were on the team and they had no idea, they were just drafted basically. (LF2, 6M Implementation Monitor Summary)”
  - “The administrators at this agency, they’ve been more of a source of barriers I would say than strategies. They really are bitter; they don’t feel like this is a project that is very well funded……..they would rather just have the State give them the money rather than”
  - “giving us money to train them. You can tell that they are skeptical about the effectiveness about the model, how much it costs, so there is definitely consensus building going on with the administrators right now. (LF3, 6M Implementation Monitor Summary)”
  - “It does not appear that the Implementation Resource Kit (IRK) materials have been used outside of intensive trainings with the consultant/trainer in ways that could address some of the clinical issues. My impression is that once the IRK trainings were over, the materials got shelved. (LF1, 6M Trainer Interview)”
- Lack of changes in agency practices
  - “This agency still thinks we know best and we are going to take care of you,”
  - “there is some resistance at this site particularly to the idea that there are things they need to change.”
  - This is an organization that has clearly decided, “We’re not gonna continue.” I think their behavior around that, their chart notes, their efforts, their supervision, have been moving away from that. They are not attending to that. So, they, knowing that they’re moving away from it, I think that they stopped putting an emphasis on it. (LF3, 24M Trainer Interview)”
  - The emphasis on the status quo was evident in the more traditional approach to services, and perceived as strengths by two low fidelity sites. As described by the program leader for LF3: “Well, there are areas of strengths, I guess, because that is the way we have always done business here.” The 24-month fidelity report capture this ‘old school philosophy’ as reported for LF3: “This group is focused on use of a 12-step informed process. Little is shared about psychiatric symptoms or coping techniques. Abstinence is expected. The group has been meeting for many years.”
- Resistance to change from practitioners
  - “Program leader stated that the use of stages in notes was not being encouraged because that “jargon” was not used throughout LF3.”
  - “They’re still doing the same old treatment that they’ve always done, and they’re not trying to incorporate some of the new stuff into there.”
- Lack of clear role delineation
  - “Who’s driving the bus is a question that really needs to be answered,”
  - “the role which should have embodied both IDDT expertise and championship was seriously unfilled.”
  - “The consultant/trainer has been aware all along of the mistakes that the agency leadership has made in the direction that the implementation has taken. He tried to steer them toward a more advantageous route but they had their own ideas. (LF1, 6M Implementation Monitor Summary)”
- Lack of time
  - “It was difficult to integrate the practice into daily work because of high caseloads (around 40) and lack of time to perform IDDT activities that are not reimbursed….and the case manager responsible for the most IDDT clients declined IDDT mini-team involvement b/c he did not feel he had time for an extra meeting every week (not surprising considering the high caseloads). (LF2, 18M Implementation Monitor Summary).”
  - “The program leader has received training, and she helps monitor the quality of the IDDT implementation. She also arranges for supervision and training. However, she is not totally empowered because of conflicts with trainer and lack of time. She does not service IDDT clients herself, and she does not have time to working to improve IDDT at LF2. (LF2, 18M Implementation Monitor Summary)”
  - “They just simply don’t have the time because of their large caseloads,”
  - “Productivity remains paramount and time for training or other learning is restricted.”
  - “staff has a lot of things on their plate outside of this project.”
- Lack of funding
  - “Comprehensive services- this is an area where deep cuts in funding have affected the program’s ability to reach high fidelity, particularly around residential and vocational services….For instance, where there are resources in the community/state, such as the IMR CCOE, the agency has been unable to mobilize the energy and time to access them. This is a product of downsizing, budget crunches, etc. that place a burden on the agency and makes it difficult for staff to tackle new projects. (LF1, 6M Implementation Monitor Summary)”
  - “In order for me to be able to do something, no matter what a great intervention technique, I have to be able to make money. If I’m not making money, then I can’t do it no matter how wonderful it is and it would almost have to be designated state by state, because Medicaid and how it funds is different in every state you go to. ..It all boils down to we want to do good clinical treatment, evidence based treatment but at the same time, that’s why we are having a problem. (LF3, 6M Program Leader Interview)
  - “LF2 administration has made it clear through their indecision about how to form the team that they do not have the money (or maybe the will) to truly implement IDDT right now. Finally, DMHA is affecting the implementation in that it is not offering the same incentives for IDDT that it does for ACT. And….voc rehab provides a disincentive by not allowing supported employment reimbursement if the IDDT client can’t be kept employed long enough. (LF2, 18M Implementation Monitor Summary)”
  - “The themes in the areas where we have low fidelity to the model usually relate to cost and revenue production.”
  - “Consultant/trainer had misgivings about the fiscal policy/philosophy of the agency administration beginning with the first interview with the CEO…..thinks that fiscal uncertainty at HF3 probably lead to turnover of team personnel and threatened stability/sustaining of the program despite its clinical soundness. (HF3, 24M Final Implementation Report)”

Knowledge

- Lack of training
  - “If you haven’t been through the training, it’s like speaking a foreign language to other clinicians. I think that they could easily pick up on it, but it’s not something they use on a regular basis. It just doesn’t make the work process easier to use in-terms that the person you’re talking to doesn’t understand. (LF3, 18M Program Leader Interview)”
  - “It is hard to squeeze it all in. There is a lack of time. They have productivity concerns that are a barrier for them to receive more training. I think they would like to learn these things, and they would like to make time, but they also do not want to lose agency incentives on their productivity. They don’t want to be punished for receiving training in the model. (LF3, 12M Implementation Monitor Summary)”
- Lack of mastery
  - “Although clinicians verbalize the principles of client choice, the lack of individual/personal goals actually incorporated into the treatment planning and documentation belies insufficient mastery of the principle.”
  - “Staff seemed to be trying to use motivational interventions in some notes, but they were still not getting it.”
  - “Team staff reported that they tailored their treatments to clients’ unique needs and goals. However, this individualized treatment was not documented in the progress notes of the charts we reviewed. In fact, some of the charts’ progress notes contained identically worded typewritten statements with blank spaces where each client’s name was written by hand. (LF2, 12M Fidelity Report)”
- Lack of clinical sophistication
  - “It speaks some to the lack of sophistication that exists not only on their team but on so many of these case management teams around the state.”
  - “These are very young case managers and are maybe in their first position and really do not have an idea about what to expect.”
  - “There was minimal understanding of these areas in previous fidelity reports. She also said she wasn’t sure that the team leader herself was grasping motivational interviewing or stage wise interventions.”
  - “This group, not only had no background in substance abuse, they had no background in anything, like 3 of their 5 case managers it was like their first job out of school, they had nothing so they needed as much mental health background and substance abuse. (LF1, 6M Trainer Interview)”
  - “Team members continue to display a range of sophistication and expertise with regard to these techniques, commensurate with their background and experience, although the level of understanding apparent in their description of interaction with clients has increased. (HF1, 12M Fidelity Report)”

Lack of priority for supervision

- “The team’s case managers receive supervision on a more informal basis,”
- “No clinicians receive structured, weekly supervision from a practitioner experienced in IDDT that is client-centered and explicitly addresses the application of IDDT to specific client situations.”
- “Supervision score =2. According to the team staff, no formal supervision time is scheduled, although staff is free to seek out supervision as needed. The recommendation is that IDDT is a model that requires a great deal of clinical skills, so all IDDT team staff could benefit from structured weekly supervision. (LF2, 24M Fidelity Report)”

**Martinez et al. 2014**

No quotes on barriers were presented in this article.

**Murray et al. 2014**

No quotes on barriers were presented in this article.

**Owczarzak et al. 2011**

Intervention level

- Lack of fit
  - “Lack of fit”
  - “After having conversations with [the prevention director] about it, the people we work with are so random. There are some people we see all the time, and some we don’t see for months, and then some people move’’ (Female, Associate Director, 9 years with agency).”
- Unique characteristics of target population
  - “Well, when you go into the jail system you can’t leave any condoms. You can’t leave literature. Initially the staff people [at the jail] wanted to sit in on a group, and that kept everybody not being open and honest. But then, they [staff people—JO] stopped coming. You can tell those women don’t want to say what’s going on with their life. They may be doing something right up in there [in jail], and they don’t want to say. And the lack of their ability to go outside. Some women that are in those institutions whereby they’re court mandated to be there, and they can’t have passes and stuff. So it’s really hard to gauge a behavioral change if they really aren’t doing anything. [Female, direct service staff,10 years with agency]”
- Time intensive
  - “We talked about it [the possibility of implementing a DEBI—JO] and talked about it, and tried to do it and just couldn’t. It was— basically we discovered that it was full time. It was a full time job just to do that program. [Another staff member] was really, really interested in trying that and doing that but 2 years later we just were like it’s, it’s, because those, those staff out there were one man. They do everything. They do needle exchange groups, bar outreach, internet outreach . . . [S]o if we had had a specific person that could be dedicated just to doing that program that would’ve been just great but [not]with the limitations we have. [Female, Associate Director, 9 years with agency]”
- Lack of desire to shift priorities
  - “I think one of the challenges, and this was a decision I had to make this week, actually, on the budget. DEBIs, because of their sustained pieces, it’s really important to have the pieces like the food and the pieces like a $40 gift card. The reality is, it’s very expensive to run them. And if you have a limited budget and you need to pay your people, you need to have pieces of your overhead in there, your indirect costs in there, all of a sudden then you’re making choices. I had to make a choice this week: do we want to run the program or do I want to have tape, or do I want to have a travel budget so that I can have staff development for my staff? I chose we need to run the program for next year. [Female, Director, 1 year with agency]”
- Intervention conflicts with organizational identity
  - “It was too focused. It’s too rigid of a focus when you are segregating down to that [young MSM—JO]. I thought back to our group and I [realized that] I’ve got more women, young women that come to group than the young men. And if we are facilitating this, if we are going to be doing this and this is going to be beneficial, it needs to be broader or it needs to be more encompassing. It needs to be a space—regardless of how you identify or what you do or how you do it—it’s going to be a place for you to go. I expressed that it was just too rigid, too rigid to sit down and have a group of 10 young women that identifies as lesbians and focus this thing directly at them because you get diversity within that group. [Male, Youth Specialist, 8 years with agency]”

Organizational level

- Staff turnover
  - “I know a challenge was staffing. Early on, we’d get somebody hired, trained and the momentum going and then [he] leaves. And then hire and trained and momentum going and now left again. This is like the third staff person in 2 years. So that’s been a huge challenge. And I think a lot of that has to do with probably the credentials of the person that we’re looking for in the position. Ideally, we like somebody that has at least started the process with AODA [alcohol and other drug abuse—JO] counseling, just the group facilitation piece. And then, the salary maybe is the issue—that we’re not paying an AODA counselor’s salary. Then they’re getting a job in an AODA field and then moving on. And then also too, our grant target has one of the primary population’s Hispanics. So getting somebody bilingual also has been a huge challenge. Bilingual individuals, especially AODA counselors, are pretty hard to keep because they’re sought after. [Male, Prevention Director, 9 years with agency]
- Make decisions regarding resource allocation, use of staff time, and agency-level priorities
  - “I do know that prevention dollars for education were cut and therefore programs . . . When I was a prevention education specialist, that program was cut. It was the HRH, High Risk Heterosexual funding we were getting. That was cut, so there was not that much money for any more education. And that was probably close to the time that maybe they just decided not to implement [VOICES/VOCES], I don’t know. Because initially, the agency has to take in the cost of whatever these programs are going to cost before the CDC or someone else gives money for it. [Female, Prevention Director, 5 years with agency]”
- Issues with funding/funders
  - “The demographics around [the agency] have been shifting. . . .We were hired to do prevention in the Latino and Latina community, and there was no mention of the fact that there still needed to be gay outreach’’

Program level

- DEBI adaptation, modification, and fidelity
  - “I don’t think anybody thinks that adapting is a bad reason, is a bad idea. I think that—this is just my impression—that the state thinks that this is something that the CDC developed [so] it’s their responsibility to come up with a Latino one . . . the state isn’t going to take it on . . . For an individual agency to take it on it would be a huge amount of work. [Female, Prevention Director, 6 years with agency]”
  - “We looked at MPowerment really closely. [My colleague] and I wanted to do it, but knew we couldn’t do it like it was written— to the extent that they wanted it done . . . [We] thought maybe we could adapt it, but we were told we shouldn’t adapt it. [Female, Associate Director, 9 years with agency]”
  - “P: Some of these other things that they suggested don’t seem 100% vital to it and they don’t fit for us anyway. So we are just going to change those but we felt like we really kept true to the core of the initiative, like the sessions and have it be group led, and building community. That was most important that we kept that. I: So you decided on your own what you saw as the core elements of this program? P: Yeah we did it and we did not go back to the people and say this is how we are going to do it. Partially because we knew they might say, ‘‘Well, that is not going to work.’’ So . . . we knew in our heads probably what they were going to say but we didn’t really want to hear them say because we knew we had to do this way anyway. We are going to have to do [it] this way and we just did it. [Male, Associate Director, 1 year with agency]”
- Lack of follow-up and accountability built into the DEBI program.
  - “P: There wasn’t a ton of follow up. I think that a lot of the assistance was one sided, us reaching out to them. There wasn’t necessarily a lot—or any, that I can remember—follow up on their end. Like, ‘‘Alright you came to this thing. What have you guys done?’’ The door was open but just us to talk to them. I: Do you think that that would’ve been helpful if they would’ve made more effort to contact you? P: Yeah, I think it would have been helpful. I think that it definitely would’ve helped with accountability if we didn’t do anything and they call us and we may feel guilty like, ‘‘Oh my gosh! We totally went to this training nine months ago and we haven’t done anything with it.’’ Also, it could just help us reinforce. . . we may have then asked other questions that didn’t feel important enough for us to call and ask this question. [Male, Associate Director of Prevention, 1 year with agency]”
- No opportunity to discuss implementation process
  - “I think it will be good if the same people who were involved in facilitating the training came [back for] meetings and discussed certain things [such as] what else can be done differently. I know that’s a lot of money—those strangers came from Colorado, but I think giving us continual education about the curriculum and how people implemented it . . . the experiences of other people doing it. That would be helpful. [Male, prevention specialist, 8 years with agency]”
- Training facilitators
  - “I think there was a lot of book knowledge . . . It sounded beautiful, but the reality was, ‘‘OK, this is going to be hard.’’ For MPowerment, it was like someone coming from California, where there is a population that we are trying to reach, and that’s a big minority number. Being MSM themselves, it was more effective [than] having someone having the book knowledge and just talking about it, saying, ‘‘This is what you do next’’ and ‘‘This is what you will do at the end.’’ And I’m thinking, ‘‘OK, it sounded nice.’’ And you get out there thinking, ‘‘I can do this,’’ but then you sit down and see, ‘‘OK, where are we going to get these people from.’’ [Male, direct service staff, 10 years with agency]”
  - “‘[The trainers] were very focused on, well, ‘This is how you spend your 40 hours. You have plenty of time to get this and this done.’ But they didn’t take into consideration that we don’t have that luxury. We are one person and have to do the testing, the driving back and forth, and the office stuff’’ (Male, prevention specialist, 5 years with agency).”

**Owczarzak 2012**

Provider perspectives on the need for EBP

- Problems with DEBI program
  - “I think it was sort of more about style than about content. I think the DEBIs are good. I think everybody needs to understand that evidence-based practice needs to be happening. You need to say why you are doing something, and you need to say what the something does to the folks that you have given it to. But the way in which it was rolled, at least the way it was perceived . . . at the time: I had co-workers and friends in the community who were at other CBOs and their perception was that their programs were devalued because they weren’t evidence based . . . And it really felt like a top down edict to a lot of people. [Female, 40s, director level, 1 year with agency]”

What works in HIV prevention

- Tension between evidence-base and their own knowledge
  - “[I]t just didn’t look feasible to me and I’m by no means an expert, please, but I kind of had a gut feeling for what would work and what wouldn’t. And that [DEBI] I was never a fan of. The people that presented to us – please, no offense – were too white to me. They were too – they didn’t seem to know my community and you are coming to tell me this is good for my community and you don’t know my community? How do you know it’s good for my community? Tell me how you came to that conclusion. So I was not – I had a lot of questions and concerns and comments. [Female, 40s, direct service provision, 14 years with agency]”
  - “For the Mpowerment [a community-level intervention for young men who have sex with men – JO], it was like someone [trainers – JO] coming from California, where there . . . [is a] population that we are trying to reach. [But, there] it’s a big minority number. And being MSM themselves it was more effective [than] someone having the book knowledge and just talking about it and saying this is what you do next, and this is what you will do at the end, and I’m thinking, ‘Ok. It sounded nice’. And you get out of there thinking, ‘I can do this’, but then you sit down and see, ‘OK, where are we going to get these people from?’ And it’s hard to get them to come in once. Three times the same people? [Female, 30s, direct service provision, 10 years with agency]”
- Realities of implementation
  - “I think a lot of [DEBIs], in my experience with them, kind of look good on paper and maybe work well in kind of a research setting, but then transferring it into an agency that’s doing the work on the streets, sometimes it just doesn’t transfer over very well . . . I don’t think the populations we serve can be as predictable as some of the DEBI’s kind of make them out to be. There’s just a huge gray area of how people respond or will respond to an intervention. [Male, 50s, director level, 9 years with agency]”
- Lack of resources for evaluation
  - “I don’t necessarily have any data or anything, but from where I sit in my office, I can see the HIV program’s door. So I see youth going in there. And not by name, obviously, but [my coworker] who does HIV tests will just kind of like anecdotally be like, ‘Yeah, so I saw 10 youth in this last 2 weeks’. [Female, 20s, direct service provision, 1 year with agency]”
  - “I think it’s very effective because you understand what you are supposed to do, and it wasn’t that difficult to start it. It was something that we had the population, and everybody wants to get together and do a social thing, even coming into the office or meeting somewhere. I think it felt like it was interesting for them because it was a time for them to meet and one-on-one, to talk about the difficulties that each of them face and to talk about risk reduction. [Female, 30s, direct service provision, 10 years with agency; emphasis added]”
- Lack of knowledge regarding evidence of effectiveness
  - “I don’t how you would measure the effectiveness . . . I guess it depends on how you look at what area of effectiveness. I mean, this program has a couple of different things that you can get from it. If you’re learning something from the program at all, any thing about your risks, it’s effective. That’s one measurement for me. Another measurable outcome for me is if you’re actually able to work toward steps toward changing your behavior, working towards any step, whether it be your first step, your second step, or your final step. That’s effective to me. Again, it focuses on baby steps. Another measurable outcome is when you can achieve a goal, whatever it is that [you] decide to achieve . . . How you overcame your own barrier . . . and brought change about in your life . . . If you have changed your thinking process in general on just how you cognitively deal with specific things . . . We’ve seen them – we’ve seen people on every stage. So I think just by them being here, it’s effective. [Female, 30s, director level, 5 years with agency]”

**Payan et al. 2017**

Planning

- Personnel capacity
  - Provider inertia
    - “I think sometimes older providers have the way they treat it, and sometimes they are stuck. They do it their way versus new ways or learning how others do it.”
- Professional development capacity
  - Lack of knowledge or experience with revised clinical guidelines
    - “It’s outdated now because we have new guidelines. I need to look at this to confirm. When we started last year, it was JNC 7 and recently, there were some changes.”
  - Lack of time to train personnel
    - “There is room for improvement in the clinics and certainly for increased provider education or documentation.”
  - Lack of consensus around medication practices
    - “There isn’t consensus around medication. If there’s guidance on that, that would be helpful.”

Implementation

- Personnel capacity
  - Management, provider, and staff retention issues
    - “We personally have had issues because of transition with leadership so it’s been hard to implement programs when there’s a constant flux in leadership so that has been one of our personal barriers.”
  - Competing responsibilities
    - “These projects are great, my own personal thing is I am frustrated because I wish I had more time . . .I wish I [had been] involved in the initial planning so we could have implemented sooner. I just don’t think I’m doing the project service with my time restriction”
- Technological capacity
  - Variable costs and delays
    - “I think that because of tremendous unforeseen problems with our EMR system, there have been long delays in the implementation of the hypertension specific decision support measures and that both of those factors have made it hard to expand the project to the volume of patients that would have made it more meaningful during the months of the study period.”

**Pemberton et al. 2012**

Implementation

- Need for greater leadership support
  - “Administration needs to learn more about MIP, what it entails, and the work conducted by staff. Lack of knowledge results in limits and less support. The organization’s leadership doesn’t place a lot of importance to MIP…there should be more orientation in this area [with] the Directors and Administration. [They] should be knowledgeable about MIP program goals and be able to understand the humanistic work that is being done.”
- Culture
  - “Even though my staff has been working with IDUs for over 8-10 years and they have expertise in terms of sitting down with a client and getting him to work through his risk-reduction goals. For the client--the main importance … is the drug and getting the drug—that’s a major barrier. I guess the other agency will tell you the same thing”
  - “They [MIP clients] will disappear but they will always come back. They may not be as structured as other people because of the drugs, but they always come back to us. MIP is client centered so you cannot make them do anything; you work with them little by little and motivate them to do better.”
  - “Activities/referrals do not always go as planned. MIP is time-consuming because of who we are working with[IDUs]. The flow from one session to the next does not always happen as planned.”
- Capacity
  - Human resource constraints
    - Inadequate or limited program staffing levels
      - “[Agency] requires more staff to run the program as intended--maybe one more staff because I need at least two outreach staff on the streets...Then I need a Counselor and Case Manager to work with MIP because that is one of the core elements of the intervention.”
      - “Although case conferences are conducted and there is some mentoring with writing clinical notes, “staging” clients, etc., staff definitely need more supervision”
      - “[Agency] model is different from what is recommended in the MIP curriculum. Each staff member does both counseling and case management—using the recommended MIP model would ease the burden.”
    - Staff skill deficits
      - “I am still convinced that MIP is better implemented by clinical staff. For example, even in how you respond to clients, there is a clinical response. I believe MIP would be more effective if implemented by people with a stronger clinical background”
      - “[I was] "learning as I go.” I would go out with my manual (MIP Implementation Manual) wherever I went and would be flipping through the pages. Am I doing this correctly? Have I covered everything? Then I gained my confidence. I still have that thing in my trunk….”
- Content
  - Meeting program fidelity (time constraints, quality, levels of service)
    - “[MIP] is a tough intervention to implement because each component can break down into multiple sessions. It is good, but it is long. When you work with IDUs you need to have an intervention that is more direct-- short --and can address all those components in a less amount of time.”
    - “We have an intervention that has 7 sessions and every session can be broken into 5-6 meetings--other sessions. Then you have a client who has a big substance abuse problem and he may not comply with the meetings that have been established. So basically…we can work with a client over 6 month period [as per MIP guidance]. During that time, let’s say the client was attending nicely and complying nicely for three months and something happens and then he disappears and the work that we have done in 3-4 months…and we are close to the end... it just disassembles in our face—so that’s a major barrier.”
    - “Meeting target number of clients as indicated in the program contract is a challenge. Some clients take 10 minutes to get through a session, and others take 3 days”
  - Implementing MIP as recommended
    - “When [original researchers] performed the study, they had a lot of case managers and staff to implement it. Now the reality of any agency working with HIV is that they don’t have a big staff; it is a few staff with various responsibilities…that is the reality in HIV[programs]—maximize the staff. When you work with an intervention as structured as this, you have to take the client to another level to really complete it…..we want to really be effective; we want to create an impact, so you have to do it right, and when you do things right it takes a little bit more time. So that’s the thing.”
    - “MIP is challenging to implement structurally. All sessions are not conducted with all clients. This is because [the] client does not follow a schedule or session flow”
    - “MIP is challenging in terms of doing it as is…..Client attendance is not reliable. If clients are no shows for over a month, you have to go back again and start from the beginning.”
    - “[We] considered the intervention presented as opposed to how it is in practice. For example, the intervention says 7 sessions but each session can take multiple contacts and run for as long as 6 months”
- Community
  - Funder specification on client eligibility for MIP
    - “The fact that qualifying MIP participants must have shared within the last 12 months has been a huge challenge. MIP staff has had to turn away individuals because of this clause.”
  - Inadequate community support/structural systems to support MIP clients
    - “So far the great challenge we have encountered is the lack of mental health programs that will take clients. It is very hard to link them for psychiatric evaluations.”
    - “Until basic needs are met client will not commit to recovery. We do not directly work on substance abuse but work with their top issues….housing, food, urgent care, etc. That is beauty of MIP.”
    - “While the community is not directly involved, they are not opposed to the MIP program either. [Agency] has a commitment to the community and is open to ideas to facilitate the effectiveness of the MIP program.”
    - “The community does not buy in as much…community has more important priorities on their plates than MIP. Also, the addiction community is transient.”
    - “[The] community sees population in a very negative way; so there is not much community support”
- Multiple categories
  - Inability to provide clients with all needed services
    - “Sometimes clients come because they have a lot of case management needs—housing, food supplies, employment—things that need to be attended quickly…and that’s part of the challenge because sometimes you cannot really resolve as you wish—because you are calling other agencies and they do not have an opening for the client—then the client may feel frustrated and he may feel that you are not really doing enough for them. Of course you try to explain to them....we always make it clear to the client that we don’t promise anything. What we can promise is that we will work with them and we are going to try our best. We never tell the clients that we are going to give them this and that because that is not a good thing. When it comes to mental health it is challenging as well to find some referrals for clients with mental health [needs].”
    - “[Agency] could improve on relationships with other agencies. Agreements are done but there is no “warm connection” with other agencies. MOA’s are on paper but the relationships are not always there or work out as intended. Sometimes we go to agencies that we know that we have agreements with and we do not have anything in common.”
  - Inability to provide clients with monetary incentives
    - “would like to see incentives used for certain unobligated things. For instance, money to supplement costs for receiving certain services where there is a small fee. For example, if you [provide] a referral for a client to seek medical care or a medical examination but they do not have the $20.00 co-pay, then they don’t go—[I]would like to see incentives used like that.”
    - “If clients do not have identification in a lot of places they cannot receive services—it is like they don’t exist—clients have to have a picture id....especially since 9-11. Clients need monetary help to get id’s.”
- MIP Program Implementation Support
  - Lack of helpfulness of funder
    - “MIP TOF was provided but no additional trainings or refreshers have been offered by the funder. We are working with what we know.”
    - “MIP TOF training, materials and documents provided by the [funder] was helpful. Since then no refresher training or new strategies have been offered to MIP staff.”
    - “Funder is not trained in intervention- "Technically I don’t know they can assist because of their own lack of knowledge. [ Funder] relies on us— the implementing agency."
    - “Funder} lacks knowledge and understanding of the intervention and
    - therefore cannot provide guidance on implementation of MIP.”

Adaptation

- Lack of knowledge on how to adapt EBIs
  - “The message for everyone trained has been that there are certain elements of the intervention that cannot be changed. You must follow the curriculum otherwise the efficacy/validity is compromised.”
  - “Depends on the core elements of the intervention. Use core elements as your rules on what is allowed with adaptation.”
  - “The core items need to be in place and not changed. There needs to be flexibility, but the essence must remain or else it becomes a totally new program.”
- No guidance on adaptation
  - “A lot of what I learned in the DEBI workshops is that the core of the program cannot be adapted—that there can be variations.. but certain things that cannot be changed.”
  - “[The] guidance is general with a focus on keeping core elements. It is not specific to populations. We are always told “don’t touch the core elements.”
  - “Some guidance on adaptation was received through trainings, but not specific to MIP”
  - “[I was] told we can adapt but not "how" to adapt.”
  - “No guidance was received on adaptation. All modifications—the terminology and forms—were done by agency staff without guidance”

**Petrescu-Prahova et al. 2016**

Implementation themes

- Lack of organizational support
  - “[The residential site] did most of the recruiting…there was no encouragement by the staff there for people to continue in the program.” Female instructor, 3 months experience
  - “…they will not allow instructors to substitute for other instructors at different Ys, which I find kind of silly…” Male instructor, 1 year experience
- Weaknesses of training
  - “I don’t think that [EF instructors] have enough knowledge after a two-day training to take on the responsibility of people’s health.” Female instructor, 2.5 years experience
- Fidelity issues
  - “I think that the fitness checks are a good idea, but it is hard to get the time to do them…” Female instructor, 2.5 years experience
  - “It’s kind of a challenge because I can’t do two things at once. Because my classes are only half an hour apart, I have to kind of do the fitness checks either before class or during class and have my sub[stitute] lead the class while I’m doing them…” Female instructor, 1 year experience
- Cost of program delivery
  - “When we came into the grant, we actually had a phenomenal opportunity to really impact those…[who] were very low income…” Male master trainer, 2.5 years experience
  - “…when we first started EnhanceFitness, I think that it was kind of hard to get the higher-ups in the [non- Y] company to buy into it. There were no grant dollars involved to get it up and running.” Female staff, 5.5 years experience
  - “I worked with [name of funder] to get this program in our [non-Y] facility, and so people didn’t have to pay… The grant ran out, and when we moved to this location there was a charge.” Female staff, 7 years experience

Maintenance themes

- Organizational structure for program delivery
  - Poor staff
    - “We went through several really, really bad instructors that the students were complaining [about]… they were going to quit because the ability of the instructors was so poor.” Female instructor, 5 years experience
  - Scheduling
    - “That was the hardest part for us scheduling-wise, because we only had so much space and so many hours that we can offer it.” Female staff, 7 years experience
- EF tasks as part of employee responsibilities
  - “There is so much paperwork that it is just ridiculous.” Female staff, 2 years experience
  - “…as long as you keep up with [the paperwork]…and turn it in regularly, it’s not a problem.” Female staff, 5.5 years experience
  - “Well, EnhanceFitness is the only one that requires paperwork. We don’t really do paperwork for any other class.” Female instructor, 1.5 years experience
- Cost for class participants
  - “For some people who are on fixed incomes, the cost of EnhanceFitness can be difficult… I think if there were more opportunities and it was cheaper for people—or it was covered by insurance like [name of program] is— it might get more people involved.” Female instructor, 1 year experience
  - “Some of my people … complained about the fact that they had to pay to take this class.” Female staff, 7 years experience
  - “I think that the Y does a fantastic job by not charging. If you’re not a member, it is a suggested donation.” Female instructor, 1 year experience

**Pinto et al. 2015**

Costs

- Staff time and precollaboration
  - “Nothing is taken off their plates”
  - “This collaboration was added on top of their initial goals and tasks”

Balancing the needs of the CBO and research

- Balance during collaboration
  - “Multiple divisions and levels require a great deal of coordination and for the CBO staff they have to add this study to the pile of other tasks.”
  - “dealing with the needs and concerns of both parties is where the challenge lies”

Communication

- Communication and future collaboration
  - “one challenge was that we weren’t able to have face time together. All communication was over email and never meeting in person is a little tough and not ideal.”

**Ramanadhan et al. 2012**

Resource constraints

- Staff capacity
  - “It’s a lot of paperwork! . . . First they said you had to do an evidence-based program. And then they said, ‘Okay, but their evidence-based evaluation isn’t enough, on top of that, you have to do ours.’ . I’ve always found it too restrictive . Participant 5 (Focus Group, Boston)”
- Material resources
  - “The evaluation . tends to be more process evaluation, than outcome evaluation . Some grants are fairly rudimentary in their outcome, in their evaluation. They just look at, ‘Did you provide the service?’ and they don’t really look at who was served or what the outcome of the person. You know, the people we serve, were they any better off at the end? Although, I think everybody would like to get to that point, nobody really knows exactly how to do it, given the limited resources to do it.—Participant 6 (Key Informant, Worcester)”

Challenges with program adaptation

- “I’m thinking like, across the country. So, if something may work really well in the Midwest, you have to think of inner-city populations of people .you’re gonna definitely find, clearly a difference in what works, scientifically, here.” —Participant 1 (Focus Group, Boston)
- We can adapt all we want, but then we have to really say, ‘Is it really . are we maintaining and keeping what it’s supposed to be?’ Cause when you translate, you kind of lose it. Not the whole thing, but you lose pieces of it.” —Participant 7 (Key Informant, Lawrence)
- We don’t change the concept, we change sort of the way maybe it’s delivered.—Participant 8” (Key Informant, Lawrence)
- “That’s all, I find that’s a challenge sometimes when . a funder kind of says . ‘Stick to the letter!’”—Participant 1 (Focus Group, Boston)”

Organizational culture

- “Sometimes, if there’s an important individual who’s interested in evidence-based programs, or who has heard about something that works, they’re more likely to do it. But I don’t think it’s part of the culture” .—Participant 6 (Key Informant, Worcester)
- I think a lot of people believe that solutions need to be developed locally. And, so, what I’ve seen more often is trying sometimes to reinvent the wheel too many times.—Participant 9 (Key Informant, Worcester)

**Thomas et al. 2014**

No quotes on barriers were presented in this article.

**Vanderpool et al. 2011**

Lack of fit

- “should be taken back where they came from. They don’t fit X County”
- “I think the biggest challenge that I felt was that when I was looking through the list…some of these things looked very specialized and were really well designed. I really didn’t feel that a lot of those specifically, not just regionally or geographically, were a good fit for our economic demographic of very low income families.”
- “I’m also very aware of the literacy issue for many of my patients. Folks don’t need all the details, they want to know how this works at my house, how can this work in my life, so I’m always looking at that angle for almost anything we do here.”

Negative perceptions

- “expected”
- “overwhelming”
- “challenging”
- “restricting”

Volunteers are unfamiliar with EBIs

- “Most of our [church] members are ‘average’ citizens who are unfamiliar with the [EBIs] concept and want to use what they believe will work.”

Difficult to adapt

- “I had to think of cramming 4 weeks of ‘Give Me Five’ programming into 45 minutes worth of time to get the one month of programming in.”
- “I felt a little distraught throughout the [adaptation] process because I didn’t think I was doing it right.”
- “I felt like it was a lot of extra work that I didn’t know if it was going to pay off in the end.”
- “…it was adapted so much to the point where it would probably be a fine line to say whether or not you could even relate the impact we had on our families to any of those particular interventions.”

Recruitment

- ‘We had an “If you build it, they will come mentality” which didn’t work’.

Funding

- “…it [the intervention] will never be a large scale success unless the money and timeframe are more realistic to more adequately show the full impact the program had on the community.’

Lack of information on EBI use in a similar context

- “We’re looking at evidence-based models now [for physical activity in the schools] …I think it gives you a definite argument as to why a program should be run, because you have the research to back you up, but again it’s hard because some of those programs you do have to tailor. There are not very much of those programs tested and run in Appalachian West Virginia. I think we have a different ball-game here…We’re all dealing with the same diseases across the US, but there is a definite culture difference here and sometimes it’s very hard to breakthrough that barrier. You have to get very creative.”

**Veniegas et al. 2009**

Preimplementation phase

- Acquiring information
  - “I didn’t realize how strict the people are to give out the information. . . . You can’t just buy it, you have to go through the training. . . . We had missed like already two trainings that were happening. . . so the next time around was going to be too late.”
- Assessing fit
  - “Evidence-based interventions don’t fit with the agency because of the complexity of the target population . . . homeless, Latino and African American, men and women . . . because of the issues that they deal with, such as language
  - barriers and culturally related issues, some of these interventions really don’t happen to apply specifically to this population”
- Preparing organization and staff
  - “We know that prevention is different for [HIV] positives and [HIV] negatives. Now you are asking us to not only adapt and tailor but to develop another curriculum to complement this.’’
- Securing technical assistance for intervention selection
  - ‘‘didn’t know how to do it either. There were no other resources available.’’

Implementation

- Securing technical assistance for implementation
  - ‘‘without having any evidence that that’s gonna make it any more or any less effective.’’
  - ‘‘We’ve tried to combine sessions because of retention, and we’ve gone through a couple of series where we’ve combined a couple of sessions together, and the clients have made it through it’’
- Conducting process evaluation
  - “We’re spending all our time trying to learn what to do, then adjust what we’re gonna do, and by the time we’re actually doing it, then our staff member leaves.’’
  - “The big barrier with retention involving adults 24, 25 and up is people work and have lives. People go on vacation. They’ll show up to one session, maybe two, but to show up for all three is really hard unless we’re providing . . . a big incentive”

Maintenance and evolution phase

- Supporting staff for continued implementation
  - ‘‘I don’t think there has been training out there that gives the staff ability to do that.’’

**Appendix S2: Extracted quotes on facilitators**

**Allicock et al. 2012**

No quotes on facilitators were presented in this article

**Amodeo et al. 2011**

No quotes on facilitators were presented in this article

**August et al. 2006**

No quotes on facilitators were presented in this article

**Belza et al. 2014**

Match with the Y mission

- “Adopting an evidence-based program fit well with the Y goals and standards and was congruent with the Y mission. The Y mission includes providing programing that helps to improve the health and fitness of older adults” (Age 30, 11 years with the Y).
- “It’s back to the spirit of mind and body of what the YMCA does (Age 33, 11 years with the Y).”
- “We knew it was an evidence-based program, one that fit well with the Y goals and standards” (Age 30, 11 years with the Y).
- I think it falls into our focus areas i.e., healthy living for seniors and social responsibility as well. We’re being responsible when we provide those types of exercise programs” (Age 29, 5 years with the Y).
- “Our strategic initiatives and our strategies roadmap for our association states very clearly that we will have a growing focus on expanding our senior membership and increasing our programing to meet the needs of the aging population”

Organizational support

- “The association office asked us if we would be interested. Because of the clientele we have, we have lots of seniors, of course I stepped up to the plate and said: “Yes, definitely let’s try this for our seniors.” That’s how we got involved” (Age 41, 14 years with the Y).
- “I think having a focus and support from junior management is important” (Age 38, 8 years with the Y).
- “I think you need to have an Executive Director or CEO really understanding what it means to deliver evidence-based programs” (Age 38, 8 years with the Y).
- “I think it’s always good thing to bring something new in. It was driven by the Y of the USA. And then also I was asked to do this by our Health and Wellness director of the main branch” (Age 51, 15 years with the Y).

Match with the target population

- “the residents [in assisted living] were good candidates because they were at a point where they had not been exercising. We could start at the beginning and see where their progress was which would not have been if we brought it into our Y and tried to offer it to our regular seniors”.
- “We had been looking for an older adult program because we have a large aging population in our community. It has been an age bracket that had been underserved at our Y” (Age 30, 11 years with the Y).
- “We are known for the fact that we offer programing that is valuable to the community and to the seniors in the community. EF is one of those programs” (Age 63, 2 years with Y-affiliated site).
- “We are in close proximity to (low income housing) and so this is a very easy place for them to come. It’s convenient for them. If we’re talking about people that are low income and don’t have money for public transportation, it makes it very easy for them to do something to take care of their healthy living” (Age 63, 2 years with Y-affiliated site).
- “It is completely appropriate for many health seekers and people who struggle with becoming more active or staying active” (Age 38, 8 years with the Y).

Financial

- “When we started it, we started with the [state department of health]… they gave us a grant basically along with other YMCAs in [the state] with all of those being downstate. They basically paid for my staff’s training and they sent us. I think that they also paid for all of our equipment. They were a huge, huge partner in this and for us being able to start EF when we did” (Age 33, 11 years with the Y).
- “The [state] contacted us and we’ve been working with them for some other programs. They offered to help with the initial training, and that’s where we learned about the program” (Age 30, 11 years with the Y).
- “We offer financial assistance. Based upon income I can give participants a certain percentage off the price of the class. And then based upon some of the grants that we have been given, I can give them even a higher percentage off. We do the best that we can to really make it happen for them. I don’t like saying no to anybody” (Age29, 5years with the Y).

Champions

- “[Champions] just do it on their own. Nobody asked them to do that (in reference to setting up fitness check areas). They just love the community that EF provides and obviously the physical benefits. They want to capture anybody that comes into class and really helps them feel that same way.”
- “I am a go-getter and if I hear something, I go after it because it is beneficial to our residents” (Age 65, 6 years with the Y-affiliated site).
- “For me, personally, it was something else for me to offer to the seniors. I absolutely love working with the active older adults” (Age 41, 14 years with the Y).
- “The more and more I learned about it, the more I loved it. I didn’t really know of any other like evidence-based programs for older adults. I really liked the pre and post-tests that they did. It just seemed like a great program” (Age 33, 11 years with the Y).
- “I told my boss about it and how I thought it would be beneficial. I told our members about it because I wanted to get them on board and get them excited. I did anything I could when the (grant sponsor) people came over. I did everything I could to promote our space” (Age 60, 7 years with Y-affiliated site).

Novelty

- “Back then [when EF was adopted] EF was kind of an experiment. There were only a few sites in the country offering it, I believe, and so I thought that it would be nice to be part of that group.”
- “I thought it would be something different, you know? I thought it would be more different and something that we could offer to our seniors (Age 41,14 years with the Y).
- “It was just something new and exciting, evidencebased. It was everything we wanted (Age51, 11 years with the Y).
- “I think it’s always good thing to bring something new in” (Age 51,16 years with theY).
- “I just wanted to have a varied program offering, and I thought this would fit…I wanted to keep the people who come here happy with our center. I want to give them a variety of things, and so I don’t want anything to be stagnant” (Age 60, 7 years withY-affiliated site).

Invitation to partner

- “The opportunity to work with and partner with an outside agency to help address another portion of our population definitely interested me.”
- “I think that I thought it looked like a great program. Our partnership at the [state department of health] was so strong. They really wanted to help the YMCAs start it” (Age 33, 11 years with the Y).
- “Sometimes the senior centers request us to do a program. That is kind of how it happened. It was just really good timing when we started EF because they were requesting that we come and do some different things. We thought it would be perfect and so it just kind of fell into place” (Age 33, 11 years with the Y).
- “I was working with a grant writer at [a university]. I was looking for something that we could get through a grant. This is the something she came up with” (Age 60, 7 years with Y-affiliated site).
- “Someone [YMCA staff member] called [my manager] and said: “We have this EF class and would you want to be our pilot program?” And she said “Absolutely, yes! That’s how it all started” (Age 51, 11 years with the Y).

Program specific facilitators

- “…an easy sale as it was proven to improve things”
- “It is evidence-based and has got solid backing. It has a proven track record and can meet the needs that are out there” (Age 30, 11 years with the Y).
- “I won’t touch anything that does not have data or an evidence-based curriculum, especially as related to chronic disease management” (Age 38, 8 years with the Y).
- “It is an incentive to bring people in when they know that you have a program that is known throughout the country. It’s a recognizable name. You are branded already” (Age 63, 2 years with Y-affiliated site).

**Collins et al. 2010**

No quotes on facilitators were presented in this article

**D'Ippolito et al. 2015**

Characteristics of Training

- “Received trainings from the person who wrote IMR.”
- “Training was excellent”
- “Good training which was thought out and planned well.”
- “The shift from communal training to individual training based on skills and experience”
- “Trainings were helpful particularly because of the use of videos.”
- “We had extensive training, a 3- or 4- part series on CBT”
- “Having an intensive external trainer.”
- “Training outreach workers to reframe questions”
- “Cultural sensitivity training.”
- “The curriculum itself is easy to understand, and could be implemented without formal training.”
- “The people who do the training are very supportive.”
- “This training spurred our staff to want other kinds of training.”

Amount and Timing of Training

- “Training is ongoing which helps to learn new model and correct implementation”
- “Continued follow-up trainings with staff”
- “Trainings so frequent it kept it fresh for staff.”
- “We go to trainings a lot.”
- “Having training at the onset of the project.”

Agency Capacity for Training

- “The ability of staff trainers to provide training.”
- “We had the funds and resources to hire an expert to provide training.”
- “The agency’s willingness and commitment to continuously train staff.”
- “Training in M.E. is offered for the entire agency, so it brings everyone on the same page.”
- “Training partners are most helpful.”

Access to and Availability of Training

- “The yearly training provided through SAMHSA. We come back energized and it reinforces that we are doing the right thing.”
- “There are a lot of agencies around to provide free or low cost trainings.”
- “Community relationships—we’re able to get in right away for trainings.”

Training Related to Specific Staff Needs

- “The clinicians were highly educated so they didn’t need much training”
- “We had a clinical supervisor on staff who was trained in ACRA”
- “Staff are well trained and well educated.”
- “My previous experience with the model through internal trainings.”
- “New staff are required to have education/training series on IDDT.”

Logistics

- “Staff went to UC San Diego conference for training.”
- “Training on IDDT that the agency provided”
- “Bringing in DBT specific training.”

**Demby et al. 2014**

No quotes of facilitators were presented in this article

**Dolcini et al. 2012**

Assess

- Fit of program
  - “It was a natural fit into the population we serve. And a large amount of our population are injection drug users. It was an easy fit. They’re not that many DEBIs that apply to injection drug users, so there wasn’t much of a choice. (ED)”

Prepare

- Having other services offered
  - “The reason that I think that we work well is that we have other supportive services in-house. And, I don’t know if all the [other funded] agencies have other supportive services as well. If they’re not, then it will be a big challenge. [Intervention name] is not a stand-alone program. You need to have other things in place. (I)”

Implementation

- Ability to adapt program
  - “Um, I think it’s gone really well. I think the trick with the intervention is really being able to adapt it and to tailor it. I think if we stuck to the curriculum very strictly it would be tough. But that there is room, I think, to aim it towards, towards the population, and I, I really feel like [the clients] are getting stuff out of it. (I)”

**Feutz et al. 2013**

No quotes on facilitators were presented in this article.

**Flores et al. 2016**

Ability to adapt program

- “There is evidence that allows us to go feeding back program execution. That feedback should be interpreted according to the characteristics of each program. The technical bidding which gives the agency are only guidelines not rules, so we can improve our intervention. It is up to each executor.”

**Gandelman et al. 2012**

No quotes on facilitators were presented in this article

**House, et al. 2017**

No quotes on facilitators were presented in this article

**Hunter et al. 2017**

No quotes on facilitators were presented in this article

**Honeycutt et al. 2012**

Leadership support

- “Pastor . . . was wonderful when it came to supporting the program. . . he was really there for me. (Site coordinator)”

Access to technical assistance

- “We’d call [TA provider] for a lot of things that we didn’t understand, as far as the implementation of programs, . . just not taking it away from the evidence-based program. (Site coordinator)”

**Kegeles et al. 2015**

HIV prevention system factors

- Planning for intervention before implementation
  - “The room they’ve ended up using is quite sterile and housed within the AIDS project.”
- Evaluation of intervention’s functioning
  - “And now they are starting to think about who they aren’t reaching…who are the people that you aren’t reaching out to…he doesn’t want it to be just about handing out flyers…he spends a lot of time getting the guys to reflect on what they are doing with the project.”

**Kimber et al. 2012**

Clinical transformation project manager

- “Having the leadership of the project manager as sort of overseeing the whole process of clinical transformation was really effective. Having that presence early on in the group meetings, that was very effective in sort of setting the stage for the process and how the process works.”

Having an inclusive change culture

- “For me, I think the most pertinent thing has been the front-line staffs’ involvement in the clinical transformation process. So, having them in the working groups, having them involved in the implementation directly, and have a real say in terms of how the interventions have come in and been developed and incorporated into their work has been imperative and significant to the process.”

Supplying needed resources

- “The management of the agency has been very supportive of our trying to manage CT as well as other commitments throughout the year.”
- “It is so easy to have meetings conflict that are equally as important as other things. That hasn’t happened because of the messaging and permission from the agency to make this (CT) a priority.”

**Lattimore et al. 2010**

No quotes on facilitators were presented in this article

**Lundgren et al. 2011**

Ability to adapt program

- “Not intentionally if we did, it depends on each person. We don't believe in cookie-cutter help, so it depends on the situation. The need is the circumstance and the individuals. I mean…I'm not going to use [EBP] completely all the way, depending on the length of recovery that an individual has, depending on their attitudes, their self esteem levels, et cetera. We haven't changed it… it's evidenced based, for God's sake. You gotta use what has been proven to be successful. But you also have to modify it for [geographic location], for the urban or the rural. I mean, so those are some of the differences I think that make it so that you have to modify it at all.”
- “So we have…made some adaptations in just how we go about the treatment, and our openness, following mostly the same principles, but we have had to make some changes throughout the process.”
- “We have over the past years …. made adaptations to the whole process and how you move people through [EBP].”

**Lundgren et al. 2012**

No quotes on facilitators were presented in this article

**Marahaj 2010**

Philosophical orientation

- Level of congruence
  - “There is a pervasive culture of respect for the client selfdirection at the agency that is very much in the spirit of recovery and consistent with the IDDT model."
  - "Focus on and respect for client choice continue to be organizing principles of treatment at the agency,"
  - "Printed materials reflect a philosophy consistent with the principles of integrated treatment, including a stagewise, client-centered approach to seamless and comprehensive SA and MI services."
  - "There's a unified philosophy that supports the principles of the IDDT EBP," while at HF3: "There is a strong foundation at the agency to reach high fidelity on this item in terms of personnel and the prevailing philosophy."
  - “I think the basic philosophy of the agency, the leadership team, the willingness to do the base work, to take the time because it is about improved outcomes for the clients. That is the bottom line here…. They're really committed to client focus. It just fits. Problem solving, solution focused, the best evidence makes sense. (HF2, 24M Program Leader Interview)”
  - “This observer finds an organization with extraordinary commitment and determination to serve dually diagnosed (DD) clients with the best services. Interviewees at multiple levels appear to embrace the integrated approach to treatment and basic principles of the model.”
  - "The agency's adoption of a recovery philosophy is very much conducive to the implementation of the IDDT model." In addition, LF3 indicates: "The program philosophy (G1) articulated at baseline assessment by all parties included elements generally supportive of evidence-based practices, in that there is a strong commitment to improving clinical treatment services to the seriously mentally ill consumer."
- Concept of culture
  - “Representatives from supervised housing communicate regularly with team members as needed, and are receptive to learning and applying IDDT principles.”
  - “Those addiction specialists are supervised by addiction managers, but they are then inserted into different teams and agencies throughout the whole agency, which involves a lot of different sites. So they are like centrally managed, but they are actually working day to day on other teams spread throughout the agency. (LF2, 6M Implementation Monitor Summary)”
  - “Clinicians report that when involved, professionals from other systems, e.g., child and family services, will attend a team or other inter-disciplinary meeting to collaborate on clients’ treatment. In general, it was reported that there is good communication and easy access among the various disciplines. (LF1, 24M Fidelity Report)”
  - “openness of the sharing of ideas and working as a team”
  - “you never feel like you’re hanging out there alone and I think the staff has really supported each other,”
  - “the strengths I think are the team itself, ”
  - “at the 12M they had an intact team that had basically all done training together and had really sort of been together fairly cohesive team.”
  - “So what’s going well however is this team is dedicated to taking care of business and so they cover for each other.”
  - “Well, the program leaders I think…. we have tried to meet regularly to be very much involved, to define our roles, to encourage communication, top down bottom. I think there is a lot of teamwork. (HF2, 18M Program Leader Interview)’

Leadership

- Role of leadership
  - “The impetus for high fidelity at this site is the agency and program leadership,” while “many of the strategies employed to increase fidelity were developed in the IDDT EBP leadership meetings.”
  - “A good foundation at the agency was due to the leadership’s knowledge and involvement.”
  - “Let’s do it in a way that helps the team understand why and what the reasons are behind it and so it encourages them and gives them the tools to do it.”
  - “The practitioners have played a part in developing and carrying out strategies. Several practitioners serve on the leadership committee. They were chosen for this committee because they showed interest in the EBP. Any practitioner that expresses interest in the IDDT EBP seems encouraged to participate in planning process. (HF2, 24M Implementation Monitor Summary)”
  - ““The team leader/program leader and the Chief Operating Officer have developed an impressive document they issued to organize team meetings and structure consumer staffing,”
  - ““there continues to be plans to develop a new comprehensive assessment protocol/tool that will incorporate IDDT principles agency-wide.”
  - “The Program Leader got it and was helping them get it before I ever showed up.”
  - ““Without a knowledgeable, available, and somewhat charismatic team leader to guide team members through everyday problems and thereby
  - “At times, the leadership team members will negotiate alternatives to a recommendation if the proposal seems unrealistic. This negotiation is significant because the leadership team has excelled with following through on the initiatives they agree to implement. Lead by the executive director, they are able to weigh what they can realistically accomplish and what will have to wait, or be modified. (HF2, 12M Implementation Monitor Summary) Chief Operating Officer who is the champion of the model and herself a skilled clinician and supervisor. Her level of involvement, particularly in the planning and implementation stages was high, and she continues to be involved at this time in a more collaborative way with the CSP Coordinator. (HF3, 6M Implementation Monitor Summary)
- Stakeholders
  - “The leadership team members seem to take the trainer’s recommendations, weigh them, and implement them when possible”
  - “The consultant/trainer was able to take what was essentially a pretty dry recitation of the model and really bring it to life in a way that connected the people very well.”
  - “he’s become very comfortable with the material and good at kind of knowing his audience and what do they need and how well these people learn best.”
  - “Involving all different parties has been real beneficial,”
  - “In ongoing steering committee or leadership team meetings, this is where we really have laid the ground work for setting up the design and structure of the IDDT program.”
  - “They are truly thinking about this steering committee as a way to involve others in the community through the implementation project.”
  - “Due to the abundance of skill and energy at the program leader level and continued Board involvement and support, the program is humming along.”
  - “They’ve got a good county board commitment and good organizational board top leadership, the top CEO is very much supportive of it and I think their providers are pretty right for it as well.”

Adaptability

- Consensus and buy-in
  - “strongly committed to build high fidelity integrated dual diagnosis programming within this agency.”
  - “The willingness to make change; the openness to feedback that the agency has had, and the enthusiasm by staff and administration.”
  - “A fidelity plan was developed following the 6M site visit and recommendations, and has driven activities to improve the program since that time.”
  - HF3’s response to improving fidelity was following “a blueprint” while “reaping the success.” HF1 acknowledged at the baseline time point: “Buy-in at both board level and provider level and some of the managers,”
  - while at the 18M time point, “everyone’s excited about the program and what we’re doing.”
  - “I don’t think without the staff buy-in and without their work and energy and imagination and practical ideas and follow-through, that’s absolutely essential.”
  - “They need to buy in that this, will overall make their job easier and will make the outcomes of their clients; they have to believe that.”
  - “Well, the first thing they did was, well, just say this was something we’re going to do, which kind of doesn’t leave people a lot of choice, you either buy into it or leave.”
  - “The consumers have played certain key roles in the IDDT implementation,” or “having people come in who tell me who are safe enough and secure enough to say this is what’s going on….that’s happening far more now than it used to.” A 24M fidelity report at HF3 reflected “client participation in goal setting and feedback.”
  - “The last steering committee that we had…. for the purpose of reviewing the six-month, fidelity was as good as any we’ve had yet with real strong consumer participation. It was the second time across the steering committees where I felt the consumers who were on the committee really stepped up and made a meaningful contribution to where we were going in terms of wanting to. (HF1, 6M Trainer Interview)”
- Changes in agency practices
  - “The most effective strategy that this agency has used so far has been to really structure the agency so that they could institutionalize change.”
  - “So it was a kind of two-fold thing between the ongoing competence with skills from the direct service staff as well as the agency structure to support the practice.”
  - “The culture of the way they do business is now built into those intensive teams: we stage, we do monthly staffing reports, we strategize by stage, we do family work, we do groups.”
  - “A significant effort has gone into the overhaul of agency documentation to incorporate IDDT language and concepts.”
  - “At the 12M point they had gone out and basically recruited staff specifically who wanted to do the model as opposed to assigning staff. They had changed the team leader role so that the program leader/team leader was going to be the sole focus for the IDDT initiative both from a management standpoint and from a team leader standpoint. They did a real nice job getting the right people in there because that group was able to talk the talk better than the training group before they’d been trained. (LF1, 12M Trainer Interview)”
- Changes in practitioner behaviors
  - “There is improvement noted in the consistency with which motivational stage is documented and interventions used, in the charts reviewed,”
  - “it’s sort of a natural growth curve I guess for them in terms of just getting better at what they do by continuing to do it.”
  - “Once case managers see consumers improve with the new programming, once they feel like they have some tools to be able to use with dual diagnosis consumers that are effective, there’s a contagious effect, and I think that’s been real positive that it builds on its own momentum so that they are more motivated to use the skills that are taught in IDDT and that helps sustain the practice. (HF2, 24M Trainer Interview)”
  - “It’s like they had a moment of clarity or something and harmonic convergence is upon them and they’re starting to do some of that stuff.”
  - “One of the things that’s emerged is that the principles of what’s going on here have sort of taken on more importance than the personalities involved,”
  - “They’re now able to engage clients that used to be seen only in emergency/crisis situations, cases that they “thought were hopeless.”
- Clear role delineation
  - “a harmonic convergence allowing people to bend their roles with an appropriate twist.”
  - “She offers the structure and he sets the clinical direction,” and “your machine, your operation here is cranking and I think it’s because each of the three of you brings something to the table.”

Knowledge

- Investment in training
  - “the managerial championing, the managerial investment because really that is the commitment to training,”
  - “every person on the team has gone to every training…..I credit a lot of their success to consistency because they’re all hearing the same thing everywhere they go.”
  - “we certainly have tried to pay attention with people coming on board, making sure that they have the orientation, and that we send them to training,”
  - “we developed a weekly study group of IDDT material in addition to formal training.”
  - “The consultant/trainer estimates that most team members have had upwards of 50 hours of IDDT training at this point. Due to this reinforcement around the skill sets such as motivational interviewing and stage-wise treatment, the team is solid clinically with respect to the model. (HF3, 6M Implementation Monitor Summary)”
  - “Senior administrators report that agency-wide cross training, workshops, and in-services seminars carrying CEUs will be used to both refresh clinicians already trained and further disseminate IDDT in the agency.”
  - “The Team Leader, dual diagnosis Program Manger and the Project Director have provided training for new hires (e.g., the new vocational specialist) and ongoing refresher and booster training both for the SAMI ACT team and to the wider agency staff. Training the new team member involved shadowing experienced staff, co-facilitating group, using the Implementation Resource Kit (IRK) manual, viewing the IRK videos, and meeting with the team leader and dual diagnosis program manager on an individual basis. (HF1, 12M Fidelity Report)”
- Mastering skills
  - “The team continues to develop, understand, and truly move toward mastery of the model’s important skill sets”
  - “Clinicians appear to have a good grasp of motivational techniques and descriptions of activities with clients show solid understanding and appropriate use of the interventions.”
  - “It was evident that many of the case managers understand the model and are able to integrate the model into their daily work.”

Priority of supervision

- “In the team meetings…..the opportunities to reinforce the language, the structure and what have you are happening ….so I think that’s just a huge piece of it and the clinicians rally by the model.”
- “All team members participate in daily 1-hour team meetings led by the team leader, receive individual supervision from either the team leader or the dual disorder program manager for 1 hour per week, meet as a team once per month for 2 hours for rotating client. The team leader receives regular structured individual supervision from the dual disorder program manager. Supervision is focused on clinicians' skills/professional development in IDDT service delivery (HF1, 12M Fidelity Report)”

**Martinez et al. 2014**

No quotes on facilitators were presented in this article

**Murray et al. 2014**

No quotes on facilitators were presented in this article

**Owczarzak et al. 2011**

Intervention level

- Fit
  - “[The SISTA intervention] fit very well. But most of the trainings [prevention interventions—JO] that I was providing at first were just single trainings, you know, the DEBI became a multisession [intervention] for one. It was more interactive in that, normally, when I’m doing an HIV 101 type of training, I am standing there just pushing information off on people. I may poll people to ask them their opinions about different things, but with DEBIs, [there are] all types of interactive exercises that people can—they don’t get lost. [Female, direct service staff, 10 years with agency]”

Organizational level

- Broad support within the organization
  - “My supervisors have always been like, ‘‘How are things going? Are we implementing it? Have you had to make any adaptations? What is working and what is not working?’’ [They] do a pretty good job of it but it’s because . . . they both have been doing HIV work in excess of 15 years, so they have been trained in the models as well. So they know they get [understand—JO] them and they follow up: ‘‘Is this working? Oh, OK.’’ [Male, direct service staff, 4 years with agency]”
  - ‘‘You gotta send people [to training]—not only your outreach staff—but you got to send managers, or people that are going to supervise’’ (Female, direct service staff, 14 years with agency).”
- Flexibility
  - “I have to say that . . . the President and the Vice-President of this here organization . . . allow me to the full capacity to be able to serve them without . . . stepping in. They do guide the program. They will oversee the parts of what it takes in that part of management, but as far as delivering with fidelity and different things, they never hinder, and they really allow me to be able to go and do my thing . . . They’re really good. [Female, direct service staff, 8 years with agency]
- Guidance
  - “I actually think that the State does a really good job with that. What has happened in the past, when we’ve had a training from the State, they will bring the trainers in . . .We go through the training. Then, a couple things can happen. [Sometimes] there is a specific group that is trained, and they’re followed on the State level and the State will support us—when we got trained for VOICES/VOCES they made extra money available to buy a variety of condoms because a lot of people can’t go out and buy a whole bunch . . . They’ve supported us in either having follow up meetings. They’ve had follow up conference calls, which can be helpful. [Our grant monitor has] CBO meetings, and every other month all of the folks who are funded by the State meet either in Madison or in the Milwaukee area. We get together and we talk about what we’re implementing, how we’re implementing it, what’s really going well, what’s not going well. Something that’s not working for us may or may not be working for somebody else. And if [our grant monitor] keeps hearing there’s something that really isn’t working for everybody, she’ll [say], ‘‘Ok, well we’ve got to do something about that,’’ versus if somebody is doing it well and then there’s one person just struggling, then that’s another opportunity to fit people together so that they have support from their other peers out doing HIV. [Female, Director, 1 year with agency]”

Program level

- Accessible facilitators
  - “We kind of checked in with the trainers and the trainers really kind of encouraged [us] to follow as closely as we could . . .We took that into mind as best we could adapt it to our population. We knew that we didn’t want to exclude women because we thought that would not send the right message from our program. So we knew we wanted to include women, so that was something that we had to stray from. We looked at the initiative and said, ‘‘This is what we see as the core—these are the most important things—and we are going to do those things.’’ Some of these other things that they suggested don’t seem 100% vital to it and they don’t fit for us anyway. So, we are just going to change those but we felt like we really kept true to the core of the initiative. [Male, Associate Director of Prevention, 1 year with agency]”

**Owczarzak 2012**

Provider perspectives on the need for EBP

- Seeing the value of EBP
  - “Once upon a time, prevention could not be measured. Now that we have these evidenced-based programs, we are seeing that prevention efforts can actually be measured and that is really important in many aspects, especially with program funding because a lot of people, in the past, or a lot of agents or government agencies didn’t necessarily want to fund prevention programs because there was nothing concrete, no concrete information to say that they did in fact work. And so I think that having evidence-based prevention programs is a milestone toward proving that prevention works. [Female, 30s, director-level, 5 years with agency]”
- Fit with agency mission and identity
  - “[Thirteen, fourteen years ago], we didn’t employ a DEBI. There was no DEBI. The woman who ran the program was called the ‘Condom Queen’, so I’m imagining that the gist of her intervention was condom demonstration. And fourteen years ago, that’s what a lot of people did. But when more energy was put into [it], we need to have an evidencebased model . . . It’s not enough for people to feel better. They have to really have gone through some change and know how to implement the change. [Female, 50s, director level, 20 years with agency]”
- Prevention efforts were worthwhile
  - “My motivation [for attending a DEBI training] really was to say, ‘Wow! Here’s a scientifically proven program that can be adopted and implemented in our area and I wanted to find out more about it . . . ’ It was scientifically based. Here’s probably one of very few programs that is actually scientifically proven to work to reduce the spread of the disease. Really, that is what we are here for and what we are doing . . . Going out and doing outreach: is that really the best use of our time? Or, here on the other hand, we have a program that’s proven so why are we not already doing that program? [Male, 30s, direct service provision, 5 years with agency]”

What works in HIV prevention

- Seeing that the program appeared to be working
  - “Well, we do risk behaviors assessments if they come back a second time and compare those; that’s like solid production of outcomes. I think that for me it’s also when they bring other people to [the program]. It’s like my peer educators that can go into the community and do presentations on safer sex. Being able to see them to do that: how many youth that they educate doing small group discussions, or larger group discussions for that matter, on safer sex, HIV and AIDS. For me that’s a success. [Male, 30s, direct service provision, 8 years with agency]”
  - “Whatever their life circumstance is, or why they’re there and sometimes it’s mostly referred or whatever, that they come in and they sit down [and in] the first few minutes [they’re like], ‘I really don’t want to be here’, or they give you excuses, [such as], ‘I need to be looking for a job. I need to be doing this. I really shouldn’t be here. What does this have to do with . . . being unemployed?’ But those are the ones that show up after that group, in session number one, that they may show up for all five sessions. I find that very rewarding for someone that initially doesn’t want to be there or has self-doubt why they’re here, and then, after telling them, we’re going to see, you’re going to see why you’re here – to learn skills. Because there’s a lot of skills building within SISTA and we’re talking about learning to be assertive versus being non-assertive or being aggressive. And that also goes along and skills for everything – jobs and anything. It doesn’t just have to be within a relationship. They learn. [Female, 50s, direct service staff, 8 years with agency]”

**Payan et al. 2017**

Initiation

- Personnel capacity
  - Quality improvement
    - “[We] have previously looked at the [hypertension] rates by site, but not by provider. We hope to do that this year as part of our 2014 quality control goals.”
- Technological capacity
  - Updated equipment to systematically capture data
    - “It’s improved the quality of patient care and preventive medicine. And it’s assisted us, the enhancement of our electronic medical record . . . in a way that will be enduring. It’s not temporary.”

Planning

- Professional developmental capacity
  - Activated medical directors who promote updated guidelines and practices
    - “Adult hypertension is a silent killer. . . . We currently identify acute and symptomatic, but we would like the clinical intervention to draw out the non-symptomatic and non-acute preventive cases and expand our existing electronic medical record capacity.”

**Pemberton et al. 2012**

Implementation of MIP

- Agency leadership support
  - “Staff have [a] strong commitment to MIP clients. There is adequate supervision and support of MIP staff. Upper management is open to staff ideas to ensure program effectiveness....The Executive Director [is] attentive to staff needs and our suggestions to improve agency services.”
  - “We get a lot of support from each other and from the Supervisor. We also have support from the Medical Director. We do both individual and group case conference two times a month. We discuss cases individually and as a group.”
  - “Our Team has free range to do what is needed for the intervention. The agency allows funds to be spent as needed and assume that they will get it back to us. Finance is never a barrier in that area.”
- Major successes with MIP Implementation
  - Positive client-level changes
    - “MIP helps client realize health is important. When clients are using drugs they forgot about themselves, so MIP helps raise awareness of needs. Eventually there are rewards. for example—when clients report that they have reduced their intake of drugs or that they are using condoms to protect themselves during intercourse, or when they say they are taking medication as mandated--things that they did not do before—to me that is an accomplishment.”
    - “What has worked well with MIP implementation is that participants are empowered and see that they have the capacity to improve their lives. MIP participants have increased self confidence.”
    - “In one case a client survived a domestic violence incident after being violently physically hurt. The participant did not relapse; she filed a police report and continued with MIP. [She] received medical and mental health services through MIP and is doing well.”
  - MIP staff capacity, commitment and teamwork
    - “There was a lot that went into implementing the program-- putting a great team together, being able to fully understand the intervention, and being committed to the fidelity…. The team did so successfully.”
    - “MIP staff work really well and [as] a unit. We are a close knit group. We have a great Coordinator that advocates for MIP staff.”
    - “We do whatever it takes. The client gets the complete service. Staff work overtime if need be and staff hours are settled later. Many times clients are accompanied to medical and service appointments. This proves to the client that [agency] has a strong commitment to assist them and will not defraud or deceive them in any way.
  - Effective intervention components benefit participants
    - “I’ve had a lot of successes in MIP. I honestly believe it is the case management piece [in MIP] because you can get counseling anywhere.….”
    - “Participants are “pleasantly surprised” with all of the MIP services that they are offered and seem to appear more secure and calmer with the process once the program is thoroughly explained to them.”
    - “It is good to know that MIP is something that we can use and that it is really working… I think that they [clients] respond to what they have been given…at different times, at different levels. [MIP] meets the client at their pace.”
    - “MIP is excellent. The participants accept it. MIP is tremendous.”
  - Use of incentives
    - “The incentives have worked well in terms of recruitment and retention of clients.”
    - “[Agency] offers significant incentives at the beginning of the program and even better at the end. [MIP clients] get gift cards of $ 20 for the 1st session (Induction session), $10 per subsequent sessions and $50 for the booster session. We give gift cards that have been suggested by participants and gift certificates to restaurants.”
    - “Clients are offered incentives for a referral; persons are screened to see if eligible for the [MIP] program. We screen them when we test them for HIV.”
    - “[Agency] sets aside weekly medical appointments for MIP participants (free of charge services). [They] receive on-site medical services--.family planning, screening and testing for HIV, comprehensive care clinic, mental health services, OB/GYN, youth services, pediatrics and dental care.”
- MIP Program Implementation Support
  - Helpfulness of funder
    - “Project Officers are always there to provide assistance every time I call. [They] always have a response and a way to help me see things or suggest ways to help me solve the issue. [The] tools are out there and the people I have had the opportunity to work with have guided me and helped me."

**Petrescu-Prahova et al. 2016**

Implementation themes

- Organizational support
  - “When someone comes in and they’re new… they [the staff] introduce them [the new person] to me… or they hand them a schedule. They’ll show them around and suggest different classes.” Female instructor, 2 years experience
  - “I started out in a small room and my class grew, and so they moved me into the gymnasium… if we need equipment, we can get it rather quickly.” Female instructor, 4 years experience
  - “…there are always substitutes available if I’m unable to teach my one class a week. The YMCA [works hard to] acquire instructors that would be adequate for the classes.” Female instructor, 5 years experience
- Instructor training
  - “…it was very well organized. The material was covered… very thoroughly. We had ample opportunity to ask questions… There was plenty of time to make it more personal for us, instead of just the blah, blah textbook.” Female instructor, 1 year experience
  - “We sat down with a PowerPoint and went over the background of EnhanceFitness… I think it’s very important to know… to be told the reasons why and the evidence behind the why was really cool.” Female instructor, 1.5 years experience
  - “What I found helpful with the whole program is that it’s structured, yet you have room for initiative. In the cardio section you can incorporate stuff from your background and from their background… when it comes to the weights, it’s structured and so you can’t go wrong.” Female instructor, 4 years experience

Maintenance themes

- Organizational infrastructure for program delivery
  - “I think that having a consistent time and space for it, we find that that really helps programs to be successful.” Female staff, 6 years experience
- Match between EF and Y Mission
  - “I’m a huge advocate of EnhanceFitness… Here at the Y we have three focus areas. We have youth development, healthy living, and social responsibility. Without a doubt, EnhanceFitness definitely hits two of those three…” Male staff, 1.5 years experience
  - “Becoming certified in EnhanceFitness allowed me to really connect with the members, especially our senior group. To me it’s very important, especially during the day here that we have our seniors enjoying their time at the Y with us. EnhanceFitness has definitely helped us do that here.” Male staff, 1.5 years experience
- Champions
  - “I’m a cheerleader for every program that I have here.” Female staff, 2 years experience
  - “There were several people who were walking advertisements for the class, they would tell one another in class the things that they were now able to do that they
  - couldn’t before.” Female instructor, 1.5 years experience
  - “They didn’t fill my position for quite some time, and so I think that not having a point person who was enthusiastic and passionate in making that a priority was a huge reason why it kind of fell through.” Female staff, 3 years experience

**Pinto et al. 2015**

Costs

- Staff time and precollaboration
  - “No one thought that this (staff time) would be a big burden so we moved forward.”
- Staff time and future collaborations
  - “the research is pretty easy going in terms of time commitment for staff”
- Financial costs
  - “this collaboration was funded by the study and the CBO just carried it out. This is helpful because the CBO doesn’t have money in the budget to do this type of collaboration.”

Benefits

- Benefits and precollaboration
  - “post-diagnosis can be very important piece in an individual’s life because it provides an important teaching moment which can help change behavior to reduce risk for recurrence and co-morbidity.”
  - “obvious benefits for patients who participated because the study promoted health and provided patient support.”
  - “the CBO funds research and research is of interest to survivors, the type of data gained would be used to better serve survivors (this is a “win–win”), the RTR volunteers would also benefit and learn how they can better assist and help survivors.”
- Benefits during collaboration
  - “this was a good opportunity to provide a program to survivors that they could participate in.”
  - “RTR program benefitted because there was deeper involvement from the volunteers and that the collaboration helped to revitalize the RTR program.”
  - “excites staff and spills over to the volunteers.”

Match with CBO's mission and goals

- Match during collaboration
  - “shared vision was the most important: both groups had vested interest in survivorship. Both parties were totally on the same page here. Both wanted to know what is in the best interest of survivors and what do they need that they don’t even know they need.”
  - “had the same values as the study. Being physically active is important to the CBO. This was key to the partnership.”
- Match and future collaboration
  - “must coincide with what they do and be in line with the goals and the mission of the CBO.”
  - “mention the appropriate focus of the research study and that it is in line with our CBO’s goals.”
- Match with champions at the CBO
  - “without this, the CBO could easily take the collaboration off their plates.”

Communication

- Communication and precollaboration
  - “communication was vital and although there was a glitch in the communications early on, this was resolved”
- Communication during collaboration
  - “The study staff was excellent in terms of communication. Communication was warm and inviting. There was always good communication, via phone, email or text.”
  - “There was good, open communication to determine the needs of the study and ways to achieve goals. It was a pleasure to collaborate.”

**Ramanadhan et al. 2012**

Program supports for implementation and adaptation

- Training
  - “One of the pieces that usually comes with an evidence-based program is that there is a training that goes along with it so you don’t have to come up with a training for people. And also, an evaluation that comes up with it. Because, coming up with your own evaluation is a killer.” —Participant 5 (Focus Group, Boston)
  - One of the grants that we’ve got.what they did was give you some intensive training on the background of how to do it . They had all the evidence for you, and gave you the tools to work with it so that you could modify and adapt it to your situation and take it. It helped.—Participant 10 (Focus Group, Worcester)

Collaborative technical assistance

- “One of the difficulties was, they have a very controlled population (but) . our population was very varied . So that was really difficult to quantify. You could see the result, but, it wasn’t as easy to quantify the way they did it in their report. So, it was a big drawback. But I had to call some of the guys . and they were very helpful to tell me what to do.” —Participant 11 (Focus Group, Worcester)”
- We (went) back to the people who developed the curriculum . (to) say, ‘‘This doesn’t work for our population, this age. We have to drop it down.’’ And they said, ‘‘You know, as long as there was this much knowledge that they already had, then you could drop it down.’’—Participant 5 (Focus Group, Boston)
- When you demand all of this sort of technical stuff, we need more assistance from the funders, from the hospitals, from scientific-based community, whatever it is.so that we can go and do the work. We need your partnership more than just, you know, plopping money on us” .—Participant 5 (Focus Group, Boston)

Perceived benefits of using proven programs

- It has the accreditation, the American Diabetes Association stamp behind it, so I certainly feel that I’m comfortable and, um, you know, that it’s got that background.” —Participant 12 (Focus Group, Lawrence)
- I think it just lends credibility to what you’re trying to do. With funders, whether it’s your…your current funder, or if you’re seeking new funding, . . there’s a credibility behind it when you can say that you use evidencebased programs.” —Participant 13 (Focus Group, Lawrence)

**Thomas et al. 2014**

No quotes on facilitators were presented in this article

**Vanderpool et al., 2011**

EBI is achievable

- ‘seemed doable or interesting’

**Veniegas et al. 2009**

Maintenance and evolution phase

- Supporting organization change and institutionalization
  - ‘‘We are continuously looking to expand the services that we offer.’’
  - Fit ‘‘with all the care services that we have, case management, education, mental health, transportation, housing, food bank.’’

**Appendix S3:** Adapted checklist for quality appraisal of cross-sectional findings

| **Items** |  | **Yes** | **No** | **Not applicable** | **Unclear** |
| --- | --- | --- | --- | --- | --- |
| *Introduction* | |  |  |  |  |
| 1 | Were the aims/objectives of the study clear? |  |  |  |  |
| *Methods* | |  |  |  |  |
| 2 | Was the study design appropriate for the stated aim(s)? |  |  |  |  |
| 3 | Was the sample size justified? |  |  |  |  |
| 4 | Was the target/reference population clearly defined? (Is it clear who the research was about?) |  |  |  |  |
| 5 | Was the sample frame taken from an appropriate population base so that it closely represented the target/reference population under investigation? |  |  |  |  |
| 6 | Was the selection process likely to select subjects/participants that were representative of the target/reference population under investigation? |  |  |  |  |
| 7 | Were measures undertaken to address and categorise non-responders? |  |  |  |  |
| 8 | Were the risk factor and outcome variables measured appropriate to the aims of the study? (e.g. could you see the questionnaire?) |  |  |  |  |
| 9 | Were the risk factor and outcome variables measured correctly using instruments/measurements that had been trialled, piloted or published previously? |  |  |  |  |
| 10 | Is it clear what was used to determined statistical significance and/or precision estimates? (eg, p values, CIs) |  |  |  |  |
| 11 | Were the methods (including statistical methods) sufficiently described to enable them to be repeated? |  |  |  |  |
| *Results* | |  |  |  |  |
| 12 | Were the basic data adequately described? |  |  |  |  |
| 13 | Does the response rate raise concerns about non-response bias? |  |  |  |  |
| 14 | If appropriate, was information about non-responders described? |  |  |  |  |
| 15 | Were the results internally consistent? |  |  |  |  |
| 16 | Were the results for the analyses described in the methods, presented? |  |  |  |  |
| *Discussion* | |  |  |  |  |
| 17 | Were the authors’ discussions and conclusions justified by the results? |  |  |  |  |
| 18 | Were the limitations of the study discussed? |  |  |  |  |
| *Other* | |  |  |  |  |
| 19 | Were there any funding sources or conflicts of interest that may affect the authors’ interpretation of the results? |  |  |  |  |
| 20 | Was ethical approval or consent of participants attained? |  |  |  |  |

Quality (circle): **High Medium Low**

Reviewer: ________________________________________________

Date: _____________________________________________________________

Study: **__________________________________________________________________________**

Rationale and comment: **____________________________________________________________________________________________________________________________________________________________________________________________________________________________________________________________________________________________________________________________________________________________________________________________________________________________________________________________________________________**

**Appendix S4:** Adapted CASP and JBI checklist

| **Checklist questions** | **Yes** | **No** | **Not applicable** | **Unclear** |
| --- | --- | --- | --- | --- |
| Was there a clear statement of the aims |  |  |  |  |
| Is a qualitative methodology appropriate? |  |  |  |  |
| Was the research design appropriate to address the research aims? |  |  |  |  |
| Was the recruitment strategy/sampling appropriate to the aims of the research? |  |  |  |  |
| Was the data collected in a way that addressed the research issue? |  |  |  |  |
| Has the relationship between researcher and participant been adequately considered? |  |  |  |  |
| Have ethical issues been taken into consideration? |  |  |  |  |
| Was the data analysis sufficiently rigorous? |  |  |  |  |
| Is there a clear statement of findings? |  |  |  |  |
| Do the conclusions drawn in the research report flow form the analysis or interpretation of the data? |  |  |  |  |

Quality (circle): **High Medium Low**

Reviewer: ________________________________________________

Date: _____________________________________________________________

Study: **__________________________________________________________________________**

Rationale and comment: **___________________________________________________________________________________________________________________________________________________________________________________________________________________________________________________________________________________________________________________________________________________________________________________________________________________________________________________________________________________**

**Appendix S5:** Less significant recommendation themes

| **Main Themes** | **Times cited** | **Citation source** |
| --- | --- | --- |
| **Recommendations for funders and collaborators** |  |  |
| Assess fit with other programming | 1 | (Belza et al., 2015) |
| Access cultural and demographic needs of the target population | 1 | (Belza et al., 2015) |
| Assess start-up and on-going costs and offsetting funding/revenue. | 1 | (Belza et al., 2015) |
| Explore adoption among majority and laggard adopters and compare to early adopters | 1 | (Belza et al., 2015) |
| Explore influence of adoption on implementation and maintenance | 1 | (Belza et al., 2015) |
| Explore policy approaches to revenue development | 1 | (Belza et al., 2015) |
| Include practitioners in the design of EBIs | 1 | (Flores et al., 2016) |
| Availability of other sources of funding and support in the external environment when implementation funding is ended. | 1 | (Hunter et al., 2017) |
| Invest in the relationship and the partnership | 1 | (Pemberton, 2012) |
| Enable and support adaptation of EBIs. | 1 | (Pemberton, 2012) |
| Emphasise benefits to TSO | 1 | (Pinto et al., 2015) |
| Continuous communication | 1 | (Pinto et al., 2015) |
| Identify champions | 1 | (Pinto et al., 2015) |
| Include TSO in needs assessment | 1 | (Pinto et al., 2015) |
| **Recommendations for research and practice** |  |  |
| Service providers also suggested the need for clearer delineation of responsibilities related to the process of DEBI adaptation, especially given the time, staffing, and resource constraints of agencies | 1 | (Owczarzak & Dickson-Gomez, 2011) |
| To common understanding of the role of evidence in program planning and delivery. | 1 | (Ramanadhan et al., 2012) |
| Communicate modifications | 1 | (Lundgren et al., 2011) |
| Develop measurement tools of fidelity | 1 | (Lundgren et al., 2011) |
| **Recommendation for practitioners** |  |  |
| Programs must consider community capacity | 1 | (Amodeo et al., 2011) |
| Willingness of organisations to engage in EB | 1 | (Lundgren et al., 2012) |
| Incorporate EBP in all levels of organisational infrastructure | 1 | (Murray et al., 2014) |
| Champions | 1 | (Petrescu-Prahova et al., 2016) |
| Invest in the relationship with funder | 1 | (Pemberton, 2012) |
| Create a culture of learning in the organisation | 1 | (Pemberton, 2012) |
| "Outreach to adolescent health care providers by program planners is recommended" | 1 | (Feutz & Andresen, 2013) |
| Understand target population | 1 | (Feutz & Andresen, 2013) |
| Introduce incentive for population to participate | 1 | (Feutz & Andresen, 2013) |
