# Supplementary material for: Barriers and facilitators to implementing evidence-based interventions among third sector organisations: a systematic review
Source: Implement Sci. 2018 Jul 30;13:103. doi: 10.1186/s13012-018-0789-7 (PMC6065156; doi:10.1186/s13012-018-0789-7)
Supplement: Supplementary file 5 — Quality of qualitative studies. (DOCX 95 kb) [file 13012_2018_789_MOESM5_ESM.docx]

**Additional file 5: Quality Appraisals of qualitative studies**

| **Study** | Was there a clear statement of the aims ? | Is a qualitative methodology appropriate? | Was the research design appropriate to address the research aims? | Was the recruitment strategy/sampling appropriate to the aims of the research? | Was the data collected in a way that addressed the research issue? | Has the relationship between researcher and participant been adequately considered?  research issue? | Have ethical issues been taken into consideration? | Was the data analysis sufficiently rigorous? | Is there a clear statement of findings? | Do the conclusions drawn in the research report flow form the analysis or interpretation of the data? | Quality assessment |
| --- | --- | --- | --- | --- | --- | --- | --- | --- | --- | --- | --- |
| Allicock et al. 2012 | X | X | X | X | X |  | X | X | X | X | Medium |
| Amodeo et al. 2011 | X | X | X | X | X |  | X | X | X | X | High |
| Belza et al. 2014 | X | X | X | X | X |  | X | X | X | X | High |
| Collins et al. 2010 | X | X | X | X | X |  |  |  | X | X | Medium |
| D’Ippolito et al. 2015 | X | X | X | X | X |  | X | X | X | X | Medium |
| Demby et al. 2014 | X | X | X | X | X |  |  |  | X | X | Medium |
| Dolcini et al. 2010 | X | X | X | X | X |  | X | X | X | X | High |
| Feutz et al. 2013 | X |  | X | X | X |  | X |  | X |  | Low |
| Flores et al. 2016 | X | X | X | X | X |  | X |  | X | X | Medium |
| Gandelman et al. 2012 | X | X | X | X | X |  | X | X | X | X | Medium |
| Honeycutt et al. 2012 | X | X | X | X | X |  | X | X | X | X | High |
| Kegeles et al. 2015 | X | X | X | X | X |  | X | X | X | X | High |
| Kimber et al. 2012 | X | X | X | X | X |  | X | X | X | X | Medium |
| Lattimore et al. 2010 | X | X | X | X | X |  |  | X | X | X | Medium |
| Lundgren et al. 2011 | X | X | X | X | X |  | X | X | X | X | High |
| Maharaj 2010 | X | X | X | X | X |  | X | X | X | X | Medium |
| Murray et al. 2014 | X | X | X | X | X |  |  |  | X | X | Low |
| Owczarzak et al. 2011 | X | X | X | X | X |  | X | X | X | X | Medium |
| Owczarzak 2012 | X | X | X | X | X |  | X | X | X | X | Medium |
| Payan et al. 2017 | X | X | X | X | X |  | X | X | X | X | High |
| Pemberton et al. 2012 | X | X | X | X | X |  | X | X | X | X | Medium |
| Petrescu-Prahova et al. 2016 | X | X | X | X | X |  | X | X | X | X | High |
| Pinto et al. 2015 | X | X | X | X | X |  | X | X | X | X | Medium |
| Ramanadhan et al. 2012 | X | X | X | X | X | X |  | X | X | X | High |
| Vanderpool et al. 2011 | X | X | X | X | X |  | X |  | X | X | Medium |
| Veniegas et al. 2009 | X | X | X | X | X |  | X | X | X | X | Medium |
