# Supplementary material for: Barriers and facilitators to implementing evidence-based interventions among third sector organisations: a systematic review
Source: Implement Sci. 2018 Jul 30;13:103. doi: 10.1186/s13012-018-0789-7 (PMC6065156; doi:10.1186/s13012-018-0789-7)
Supplement: Supplementary file 6 — Quality of survey studies. (DOCX 91 kb) [file 13012_2018_789_MOESM6_ESM.docx]

**Additional file 6: Quality Appraisals of Quantitative Studies**

| **Quality items** | House et al. 2017 | Hunter et al. 2017 | Lundgren et al. 2012 | Martinez et al. 2014 | Thomas et al. 2014 |
| --- | --- | --- | --- | --- | --- |
| The aims/objectives of the study were clear | X | X | X | X | X |
| The study design was appropriate for the stated aim(s) | X | X | X | X | X |
| The sample size was justified | X | X |  | X | X |
| The target/reference population was clearly defined (It clear who the research was about?) | X | X | X | X | X |
| The sample frame was taken from an appropriate population base so that it closely represented the target/reference population under investigation | X | X | X | X | X |
| The selection process was likely to select subjects/participants that were representative of the target/reference population under investigation | X | X | X | X | X |
| Measures were undertaken to address and categorize non-responders |  |  |  |  | X |
| The risk factor and outcome variables were measured appropriately to the aims of the study (e.g. the questionnaire could be seen) |  | X | X | X | X |
| The risk factor and outcome variables were measured correctly using instruments/measurements that had been trialled, piloted or published previously? |  | X | X | X |  |
| It is clear what was used to determined statistical significance and/or precision estimates? |  | X | X | X | X |
| The methods (including statistical methods) were sufficiently described to enable them to be repeated |  | X | X |  | X |
| The basic data were adequately described | X | X | X | X | X |
| The response rate does not raise concerns about non-response bias |  | X | X |  |  |
| If appropriate, information about non-responders was described |  |  |  |  |  |
| The results were internally consistent | X | X | X | X | X |
| The results for the analyses described in the methods were presented | X | X | X | X | X |
| The authors’ discussions and conclusions are justified by the results | X | X | X | X | X |
| The limitations of the study were discussed | X | X | X | X | X |
| Funding sources or conflicts of interest did not likely affect the authors’ interpretation of the results | X | X | X | X | X |
| Ethical approval or consent of participants attained |  | X | X | X | X |
| **Final quality assessment** | **Low** | **Med** | **Med** | **Med** | **Med** |
